# The Enteric Bacterium *Enterococcus faecalis* Elongates and Incorporates Exogenous Short and Medium Chain Fatty Acids Into Membrane Lipids

**DOI:** 10.1111/mmi.15322

**Published:** 2024-10-08

**Authors:** Qi Zou, Huijuan Dong, John E. Cronan

**Affiliations:** ^1^ Department of Microbiology University of Illinois at Urbana‐Champaign Urbana Illinois USA; ^2^ Department of Biochemistry University of Illinois at Urbana‐Champaign Urbana Illinois USA

**Keywords:** acyl‐phosphates, elongation, phospholipids, short chain fatty acids

## Abstract

*Enterococcus faecalis* incorporates and elongates exogeneous short‐ and medium‐chain fatty acids to chains sufficiently long to enter membrane phospholipid synthesis. The acids are activated by the *E. faecalis* fatty acid kinase (FakAB) system and converted to acyl‐ACP species that can enter the fatty acid synthesis cycle to become elongated. Following elongation the acyl chains are incorporated into phospholipid by the PlsY and PlsC acyltranferases. This process has little effect on *de novo* fatty acid synthesis in the case of short‐chain acids, but a greater effect with medium‐chain acids. Incorporation of exogenous short‐chain fatty acids in *E. faecalis* was greatly increased by overexpression of either AcpA, the acyl carrier protein of fatty acid synthesis, or the phosphate acyl transferase PlsX. The PlsX of *Lactococcus lactis* was markedly superior to the *E. faecalis* PlsX in incorporation of short‐chain but not long‐chain acids. These manipulations also allowed unsaturated fatty acids of lengths too short for direct transfer to the phospholipid synthesis pathway to be elongated and support growth of *E. faecalis* unsaturated fatty acid auxotrophic strains. Short‐ and medium‐chain fatty acids can be abundant in the human gastrointestinal tract and their elongation by *E. faecalis* would conserve energy and carbon by relieving the requirement for total *de novo* synthesis of phospholipid acyl chains.

## Graphical abstract

The enteric bacterium *Enterococcus faecalis* incorporates short fatty acids from the medium and elongates the chains to lengths suitable for membrane lipids. Unlike long chains the short chains do not down regulate fatty acid synthesis.

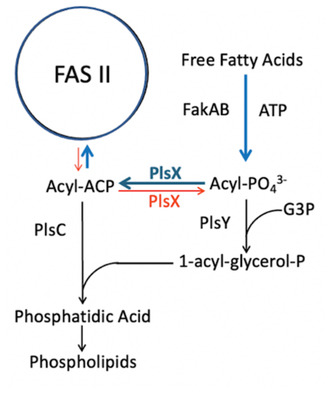

## 1 Introduction

Fatty acid biosynthesis, one of the most ubiquitous cellular metabolic pathways, provides precursors for cell membrane phospholipid synthesis, secondary metabolite synthesis, signaling molecules and protein post‐translational modifications (Beld, Lee, and Burkart 2015). This process requires the Type II fatty acid synthase (FAS II), a set of discrete enzymes, found in bacteria, mitochondria, and plant plastids (Beld, Lee, and Burkart 2015). Acyl carrier protein (ACP), the carrier of the elongating fatty acyl chain not only plays a crucial role in fatty acid and phospholipid synthesis but also has a role in incorporation of exogenous fatty acids (Yao and Rock 2013, 2017; Zhu et al. 2019; Zou et al. 2022).

**FIGURE 1:**
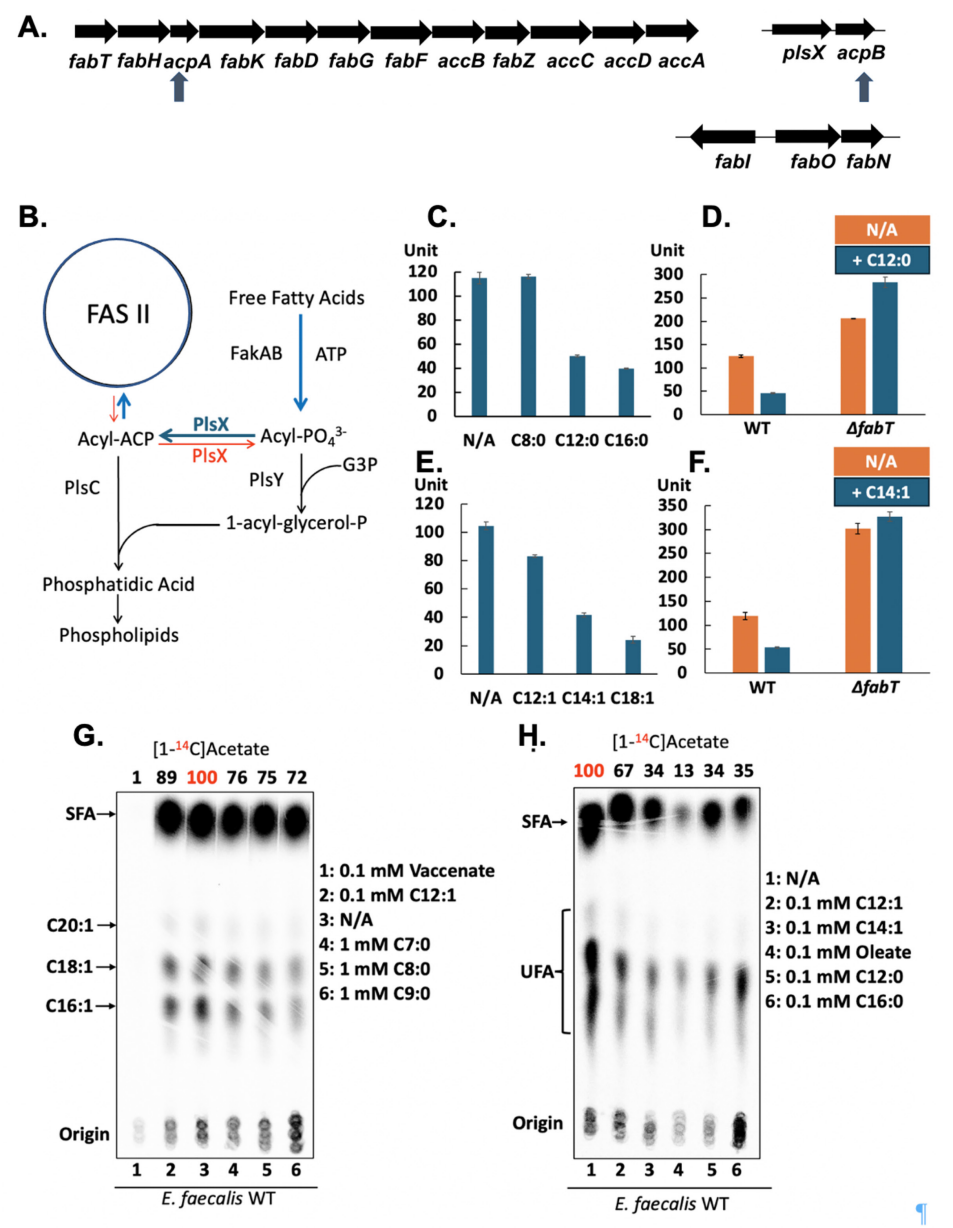
Effects of exogenous short chain fatty acids on *de novo* fatty acid synthesis and incorporation in *E. faecalis*. (A) The *E. faecalis* fatty acid metabolism operons. The two *acp* genes are marked with arrows. The first gene of the large operon is *fabT* which encodes the FabT transcriptional repressor. This results in autoregulated expression of FabT. FabT also regulates the *fabO fabN* operon plus the divergent *fabI* gene but not the *plsX*‐*acpB* operon which functions in fatty acid uptake. (B) Phospholipid synthesis and incorporation of exogenous fatty acids in the *Lactobacillales*. (C–F) Expression of β‐galactosidase driven by the *E. faecalis fabT* promoter in the presence of (C and D) exogenous saturated or (E and F). unsaturated fatty acids in the *E. faecalis* FA2‐2 wild‐type strain. (D and F) Expression of β‐galactosidase driven by the *E. faecalis fabT* promoter in the presence of (D) lauric acid or (F) *cis*‐5‐tetradecenoic acid in the *E. faecalis* wild‐type and *∆fabT* strains. (G) The effects of hexanoic acid, heptanoic acid, octanoic acid or *cis*‐5‐dodecenoic acid on *de novo* phospholipid fatty acyl chain synthesis in the *E. faecalis* wild‐type strain. (H) The effects of lauric acid or *cis*‐5‐tetradecenoic acid on *de novo* phospholipid fatty acyl chain synthesis by the *E. faecalis* wild‐type strain. The red numbers denote the [^14^C]acetate incorporation by the wild‐type strain in the absence of a supplemented fatty acid.

Phospholipids are essential structural components that form the membrane bilayer boundary between the cell cytoplasm and the extracellular environment (Yao and Rock 2013, 2017; Zhu et al. 2019; Zou et al. 2022). The key intermediate in the synthesis of phospholipids is phosphatidic acid, the formation of which requires two successive acylations of the glycerol‐3‐phosphate (G3P) backbone (Yao and Rock 2013, 2017). In addition to *de novo* fatty acid synthesis, bacteria utilize free fatty acids from the environment as donors of acyl chains (Yao and Rock 2013; Yao and Rock 2017). In gram‐positive bacteria, environmental fatty acids are activated by the two‐component fatty acid kinase system (Fak) to form acyl‐phosphates. The Fak system is composed of two components, the dimeric FakA fatty acid kinase and two copies of a FakB fatty acid binding proteins of which *E. faecalis* has four different protein species. The acyl‐phosphates are either utilized by the PlsY G3P acyltransferase to acylate the *sn*‐1 position of G3P or converted to acyl‐ACP by the PlsX phosphate acyltransferase to acylate the *sn*‐2 position of lysophosphatidic acid by PlsC (Figure 1B) (Gullett et al. 2019; Parsons et al. 2014a, 2014b; Shi et al. 2022). As will be discussed below *E. faecalis* is unusual in that it encodes two acyl carrier proteins (ACPs) rather than the single ACP found in most bacteria (Cronan 2023). AcpA is required for *de novo* fatty acid synthesis (Dong and Cronan 2022a) whereas AcpB functions in the uptake of exogenous fatty acids (Zhu et al. 2019). Both *E. faecalis* ACPs act in the regulation of fatty acid synthesis after conversion to acyl‐ACP species having long acyl chains. Regulation is due to the FabT transcriptional repressor that when complexed with a suitable acyl‐ACP binds the promoter of the large *fabT‐accA* fatty acid synthesis operon and the promoters of the *fabI* and *fabON* operons (Figure 1A) (Hays et al. 2021; Lambert et al. 2022; Zhu et al. 2013; Zou et al. 2022). The *fabT is* the first gene of the *E. faecalis fabT‐accA* operon and hence its expression is autoregulated (Hays et al. 2021; Zhu et al. 2019; Zou et al. 2022).

*E. faecalis* is an extraordinarily hardy bacterium that readily adapts to widely diverse habitats. In humans, this bacterium is primarily a commensal gastrointestinal occupant (Fiore, Van Tyne, and Gilmore 2019; Garcia‐Solache and Rice 2019). *E. faecalis* is unusually resistant to common antiseptics and disinfectants, as well to UV radiation, starvation and desiccation and thus survives very well in hospital settings (Fiore, Van Tyne, and Gilmore 2019; Garcia‐Solache and Rice 2019). The ability to tolerate these conditions is key to its pathogenicity because it is not a highly virulent organism, rather it is largely an opportunistic pathogen (Fiore, Van Tyne, and Gilmore 2019; Garcia‐Solache and Rice 2019). *E. faecalis* readily acquires mobile elements that encode resistance to a large multiple antibiotics (mobile elements can occupy as much as 25% of a *E. faecalis* genome) and these resistance elements can be considered a type of virulence determinant. The major sites of infection are surgical procedures especially gastrointestinal and dental (root canal) surgery and *E. faecalis* has mainly been studied in this context (Fiore, Van Tyne, and Gilmore 2019; Garcia‐Solache and Rice 2019). Study of the metabolism and physiology of this bacterium has been hampered by the lack of a generally applicable chemically defined medium. Hence, several different complex growth media are used which complicates comparisons among literature reports. Aside from the work reported in this report and prior publications from this laboratory, studies of fatty acid uptake in *E. faecalis* have been limited to long‐chain acids. Fozo and coworkers (Saito, Harp, and Fozo 2017; Woodall, Fozo, and Campagna 2023; Woodall et al. 2021) have monitored incorporation of exogenous fatty acids of chain lengths > C12 into membrane lipids and the effects of fatty acid supplementation on stress conditions and cell morphology. Hays et al. (2021) deleted the large fatty acid operon (Figure 1A) from the *E. faecalis* genome. Growth of this mutant strain was supported by a high concentration of human serum, which contains a mixture of long‐chain fatty acids. These investigators showed that addition of serum resulted in severely decreased transcription of the fatty acid synthesis genes as expected from prior work with oleic acid supplementation (Zhu et al. 2019).

We report that the *E. faecalis* incorporates and elongates exogenous short‐ and medium‐chain fatty acids. Increased expression of either AcpA or the PlsX phosphate acyltransferase in *E. faecalis* increased utilization of both short‐ and medium‐chain fatty acids although this varied with the acyl chain. The incorporation of short‐ and medium‐chain fatty acids by *E. faecalis* relies on levels of coordination between fatty acid synthesis and the PlsX acyltransferase. Upon conversion to acyl‐ACPs exogenous short‐chain fatty acids elongated to sufficient chain length repressed *E. faecalis de novo* synthesis of fatty acids through interaction with the FabT repressor. The PlsX of *L. lactis* was found to be superior to the PlsX of *E. faecalis* in the incorporation of short‐ and medium‐chain fatty acids.

## 2 Results

### 2.1 Exogenous Fatty Acids of Sufficient Chain Length Repress *E. faecalis* Fatty Acid Synthesis

The first question was whether exogenous short‐chain fatty acids repress *de novo* fatty acid synthesis as observed with longer‐chain acids (Hays et al. 2021; Zhu et al. 2013; Zou et al. 2022). If so, this would indicate that the acids had been converted to acyl‐ACP species, which are the regulatory ligands. Unlike most bacteria, *E. faecalis* and closely related bacteria have two ACP proteins. AcpA is essential for fatty acid synthesis and AcpB does not substitute for the loss of AcpA (Dong and Cronan 2022a; Hays et al. 2021). The primary role of AcpB is fatty acid uptake (Zhu et al. 2019; Zou et al. 2022). The binding of acylated species of both ACPs activates DNA binding of the FabT fatty acid synthesis transcriptional repressor (Zou et al. 2022; Hays et al. 2021). The structures of both ACPs have been determined by NMR (Yoo et al. 2024). Both are highly stable proteins having typical ACP helical structures although AcpA lacks the typical short helix 3 which is likely the reason it cannot replace the AcpP of *E. coli* (De Lay and Cronan 2007).

A direct test of fatty acid regulatory ability was assay of β‐galactosidase (LacZ) synthesis driven by the autoregulated *fabT* promoter (Zou, Dong, and Cronan 2023). The *fabT* gene is the first gene of *E. faecalis fab* gene cluster and encodes the transcriptional regulator FabT that requires acyl‐ACP as the ligand for repression (Zhu et al. 2019; Zou et al. 2022). Octanoic acid (C8:0), decanoic acid (C10:0) and undecanoic acid (C11:0) each failed to repress β‐galactosidase production driven by the *fabT* promoter whereas lauric acid (C12:0) supplementation resulted in an ~2‐fold repression (Figure 1C,D; Figure S1A,B). Supplementation with *cis‐*5‐dodecenoic acid (C12:1) gave an ~30% reduction in β‐galactosidase whereas the presence of *cis*‐5‐tetradecenoic acid (C14:1) resulted in an ~60% decrease (Figure 1E,F). Consistent with previous results (Zou et al. 2022), repression of β‐galactosidase expression was detected in cultures of the wild‐type strain supplemented with palmitic acid (C16:0) (~3‐fold) or oleic acid (*cis*‐9 C18:1) (~4‐fold) (Figure 1C,E). However, inhibition by lauric acid or *cis*‐5‐tetradecenoic acid was blocked by deletion of *fabT*, which relieved repression of *de novo* synthesis (Figure 1D,F). Similar repression of β‐galactosidase production from the *fabT* promoter by lauric acid (C12) or *cis*‐5‐tetradecenoic acid was also observed in the *E. faecalis ∆acpB* strain (Figure S1C,D). The repression levels for *fabI* and *fabO*, the other two FabT regulated promoters (Figure S1E,F) (Zou et al. 2022) were similar. Hence, as expected following their conversion to acyl‐ACPs of sufficient chain length to bind FabT, exogenous fatty acids repressed expression of the *E. faecalis fab* genes (Zhu et al. 2019).

Labeling with [1‐^14^C]acetate measured *de novo* synthesis of phospholipid fatty acyl chains in the presence of various exogenous fatty acids. Supplementation with heptanoic acid (C7:0), octanoic acid (C8:0) or nonanoic acid (C9:0) had little or no effect on *de novo* fatty acid biosynthesis in the *E. faecalis* wild‐type strain (Figure 1G). In contrast, the C18 unsaturated fatty acids, oleate or *cis‐*vaccinate, or palmitate, the C16 saturated fatty acid, essentially abolished *de novo* synthesis (Figure 1G,H) as reported previously (Zhu et al. 2019). The presence of the shorter unsaturated acid *cis*‐5‐dodecenoic acid (C12:1) failed to repress *de novo* fatty acid synthesis (Figure 1G,H). However, consistent with the above β‐galactosidase assays, supplementation with lauric acid (C12:0) or *cis*‐5‐tetradecenoic acid (C14:1) resulted in 3‐fold repression in *de novo* synthesis of fatty acids in the wild‐type strain (Figure 1H). Similar inhibition by lauric acid or *cis*‐5‐tetradecenoic acid was also observed in the *E. faecalis ∆acpB* strain (Figure S2 lanes 3 and 4). Note that elongation of short chains would result in somewhat decreased [1‐^14^C]acetate incorporation since the phospholipid acyl chains would contain fewer labeled acetate units.

### 2.2 Are Short Chains Activated by the *E. faecalis* Fatty Acid Kinase and Transferred to ACP?

**FIGURE 2:**
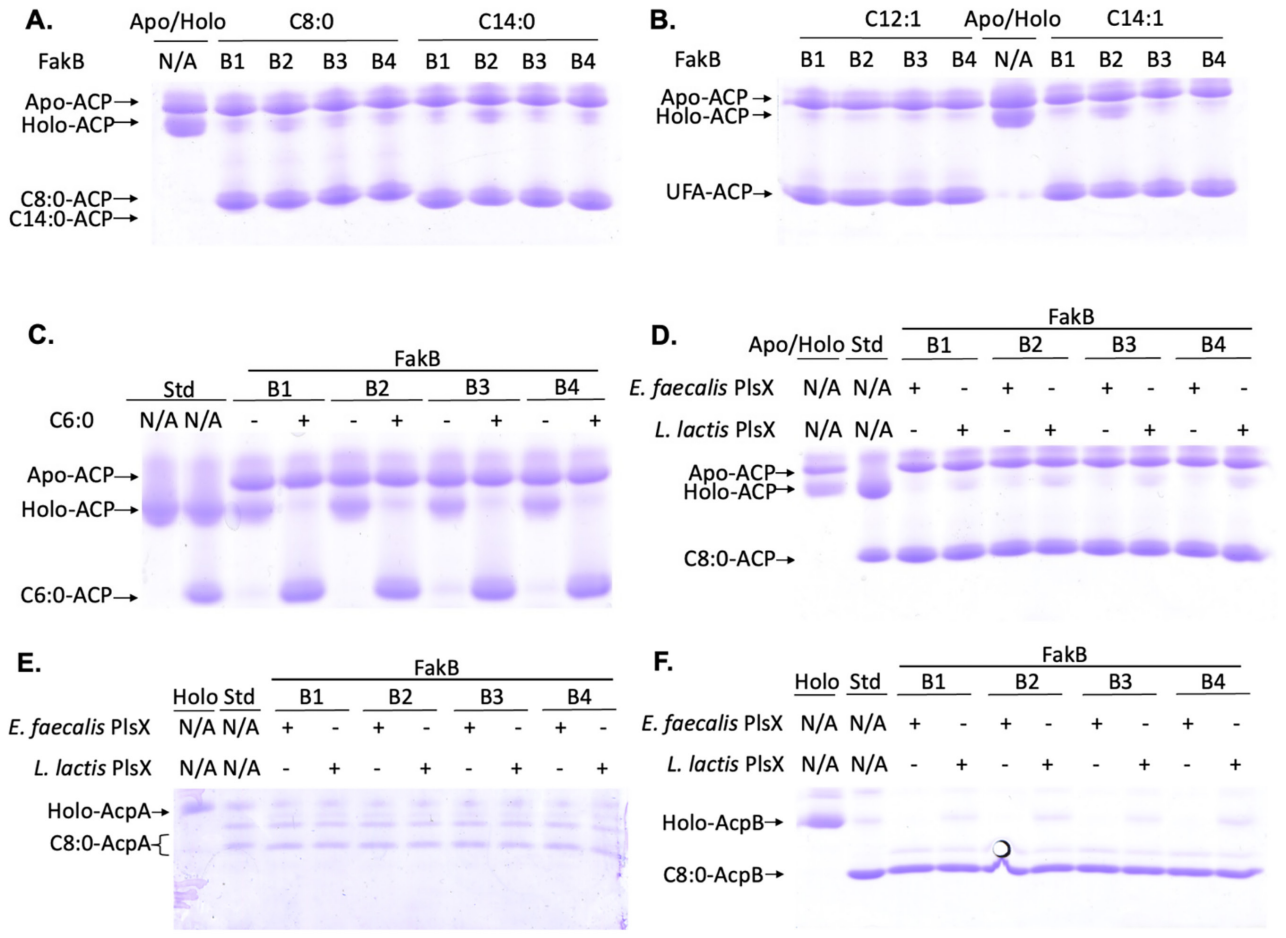
The *E. faecalis* FakA fatty acid kinase activates and PlsX transfers short‐chain fatty acids. The reactions were done as described previously (Zou et al. 2022). (A and B) Acylation of ACP with (A) short‐chain saturated fatty acids or (B) unsaturated fatty acids via the *E. faecalis* Fak and PlsX reactions. (C) Synthesis of hexanoyl‐ACP through the *E. faecalis* Fak and PlsX reactions. (D) Fak‐based synthesis of octanoyl‐ACP by transfer from the acyl‐phosphate by *E. faecalis* PlsX or *L. lactis* PlsX. The *E. coli* hexanoyl‐ACP and octanoyl‐ACP standards were synthesized from hexanoyl‐CoA and octanoyl‐CoA, respectively, by the Sfp 4′‐phosphopantetheinyl transferase. (E and F) Octanoylation of *E. faecalis* (E) AcpA or (F) AcpB by the Fak‐PlsX reactions. The *E. faecalis* octanoyl‐AcpA and octanoyl‐AcpB standards were synthesized from octanoyl‐CoA by the *B. subtilis* Sfp 4′‐phosphopantetheinyl transferase.

The inability of short‐chain acids to inhibit or regulate *de novo* synthesis could be explained by failure of these acids to be phosphorylated by the *E. faecalis* fatty acid kinase (FakAB) system due to chain length specificity of the proteins (Parsons et al. 2014a, 2014b; Zou et al. 2022). Moreover, PlsX may not accept short‐chain acyl phosphates. Either of these would eliminate the PlsX‐catalyzed synthesis of the short‐chain acyl‐ACPs required for elongation. We tested these possibilities by in vitro assay of FakAB‐PlsX activity on short‐ and medium‐chain fatty acids (Parsons et al. 2014a, 2014b; Zou et al. 2022) (Figure 2A–F). In this assay, a FakB binding protein must bind the acyl chain and present the carboxyl group to the FakA fatty acid kinase for acyl‐phosphate synthesis. The acyl‐phosphates are then converted to acyl‐ACPs by the PlsX acyltransferase. Conformation‐sensitive gel electrophoresis showed almost complete conversion to acyl‐ACPs of all fatty acids tested. The acyl ACP thioesters of the C6:0, C8:0, C14:0, C12:1, and C14:1 species were all produced (Figure 2A–C). Like *E. faecalis* PlsX, *L. lactis* PlsX produced C8:0‐ACP in the Fak‐PlsX assay (Figure 2D). Both *E. faecalis* AcpA and AcpB were converted to octanoyl thioesters by the Fak‐PlsX reactions in vitro (Figure 2E,F). In summary, these data indicated that the *E. faecalis* fatty acid kinase efficiently activated short‐ and medium‐chain fatty acids thereby allowing their PlsX‐catalyzed conversion to acyl‐ACPs. In addition, the *E. faecalis* FakAB system synthesized the acyl‐ACP thioesters of *trans*‐2‐octenoic acid (C8:1) or *trans*‐2‐decenoic acid (C10:1) known enoyl intermediates of fatty acid synthesis (Figure S3).

### 2.3 Short‐Chain Acids Are Elongated by *E. faecalis*

The in vitro data above indicating the conversion of short‐chain acids to ACP thioesters argued that these thioesters should enter the fatty acid synthesis cycle. This would allow elongation to acyl chains sufficiently long for incorporation into phospholipids by the PlsY and PlsC acyltransferases. We expected that only AcpA thioesters would be elongated since *∆acpA* strains are completely defective in fatty acid synthesis and AcpB overexpression is unable to restore synthesis (Dong and Cronan 2022a). This was further supported by the fact that *E. faecalis* FabD malonyl‐CoA‐ACP transacylase preferred AcpA rather than AcpB in converting malonyl‐CoA to malonyl‐ACP the substrate for fatty acyl chains synthesis (Figure S4A). Lack of AcpA resulted in an inability to elongate exogenous *cis*‐7‐hexadecenoate to *cis*‐9‐octadecenate (oleate) as shown by analysis of phospholipid acyl chains (Figure S4B).

**FIGURE 3:**
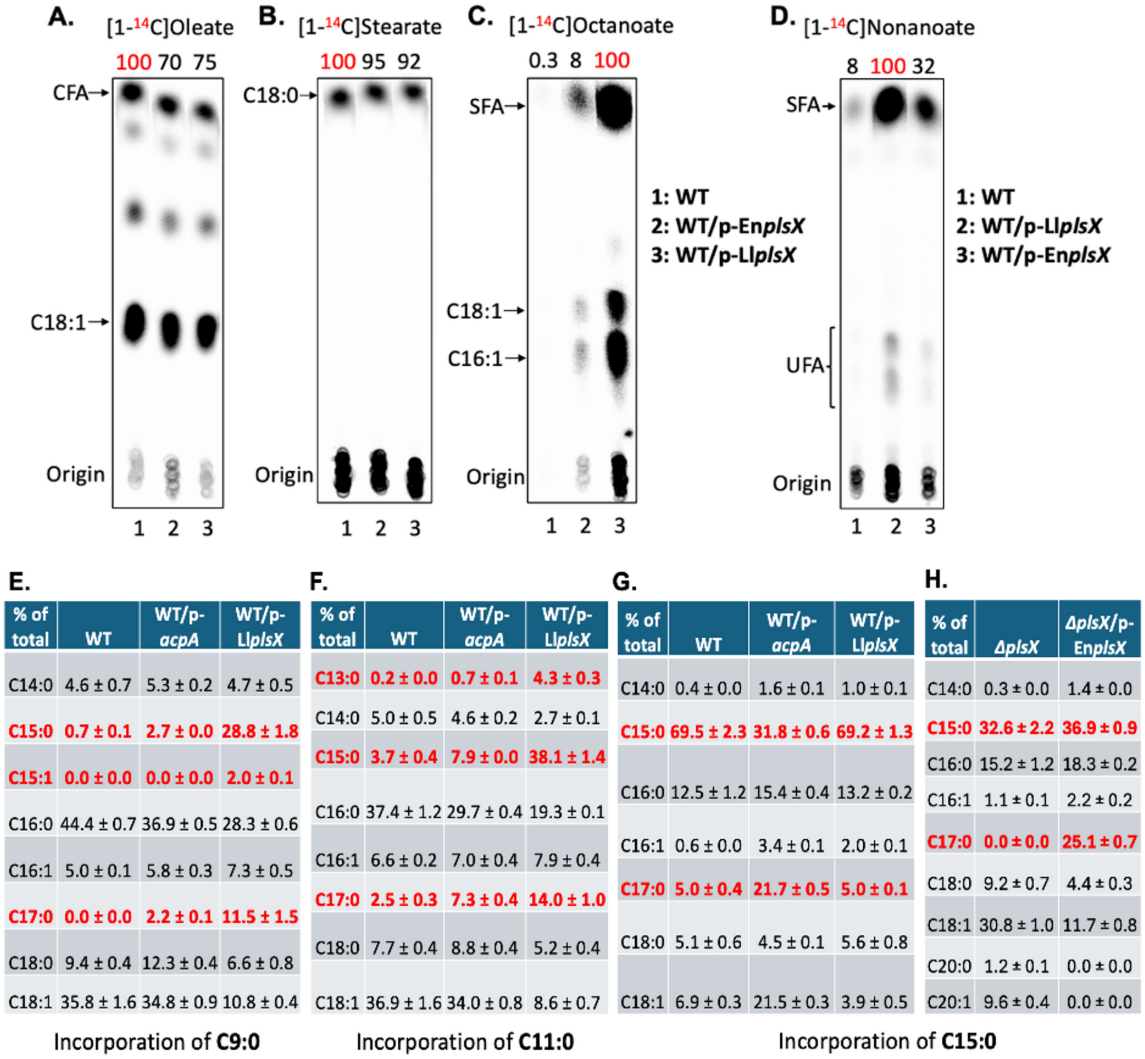
Overexpression of *E. faecalis* PlsX or expression of *L. lactis* PlsX increases incorporation and elongation of short‐ and odd‐chain fatty acids. Panels (A–C) have the same lane designations whereas Panel D has its own. (A–D) Incorporation of (A) [^14^C]oleate, (B) [^14^C]stearate, (C) [^14^C]octanoate or (D) [^14^C]nonanoate by the *E. faecalis* wild‐type strain overexpressing *E. faecalis* PlsX or expressing *L. lactis* PlsX. The numbers above the lanes are the radioactive label incorporation values relative to the value (100) for the wild‐type strain (A and B) or wild‐type strain expressing *L. lactis* PlsX (C and D). (E–G) GC‐MS analysis of incorporation and elongation of (E) nonanoic acid (C9:0), (F) undecanoic acid (C11:0), or (G) pentadecanoic acid (C15:0) by the *E. faecalis* wild‐type strain overexpressing AcpA or expressing *L. lactis* PlsX. (H) Incorporation and elongation of pentadecanoic acid (C15:0) by *E. faecalis ∆plsX* and its complemented strains. Note the lack of elongation to C17:0 in the *∆plsX* strain. Panels A–C have the same lane designations. In Panel E, the nonanoate was supplied at 1 mM.

Direct proof of elongation was provided by use of [1‐^14^C]‐labeled acids. *E. faecalis* lacks a β‐oxidation pathway hence the labeled acids are incorporated intact as previously demonstrated with deuterated octanoate (Morgan‐Kiss and Cronan 2008) and more recently with [U‐^13^C] oleate (Woodall, Fozo, and Campagna 2023). Unlike incorporation of [1‐^14^C]oleic acid or [1‐^14^C]stearic acid (Figure 3A,B), incorporation of [1‐^14^C]octanoic acid or [1‐^14^C]nonanoic acid was marginal in the wild‐type strain unless PlsX was overexpressed (Figure 3C,D). Note that [1‐^14^C]octanoic acid was incorporated into both saturated and unsaturated species (Figure 3C) whereas [1‐^14^C]nonanoic acid mainly labeled saturated species (Figure 3D). Interestingly, *L. lactis* PlsX was considerably more efficient than *E. faecalis* PlsX in incorporation of [1‐^14^C]octanoic acid and [1‐^14^C]nonanoic acid (Figure 3C,D) whereas PlsX overexpression gave no increase in incorporation of [1‐^14^C]oleic acid or [1‐^14^C]stearic acid (Figure 3A,B). Consistent with the radiolabeled fatty acid data, *L. lactis* PlsX expression resulted in higher levels of odd numbered phospholipid acyl chains from elongation of nonanoic acid and undecanoic acid by GC‐MS analysis (Figure 3E,F). However, this was not the case for pentadecanoic acid (C15:0), which is directly transferred to phospholipids (Figure 3G). Overexpression of AcpA weakly increased the levels of nonanoic acid and undecanoic acid incorporated into phospholipids of the *E. faecalis* wild‐type strain (Figure 3E,F) and markedly increased elongation of pentadecanoic acid (Figure 3G). Deletion of PlsX resulted in decreased incorporation into phospholipids since the only acyltransferase substrate was pentadecanoyl‐phosphate for acylation of the *sn‐*1 position. Restoration of PlsX allowed synthesis of pentadecanoyl‐ACP for acylation of the *sn*‐2 position and elongation (Figure 3H).

### 2.4 Effects of Simultaneous Overexpression of AcpA and PlsX

**FIGURE 4:**
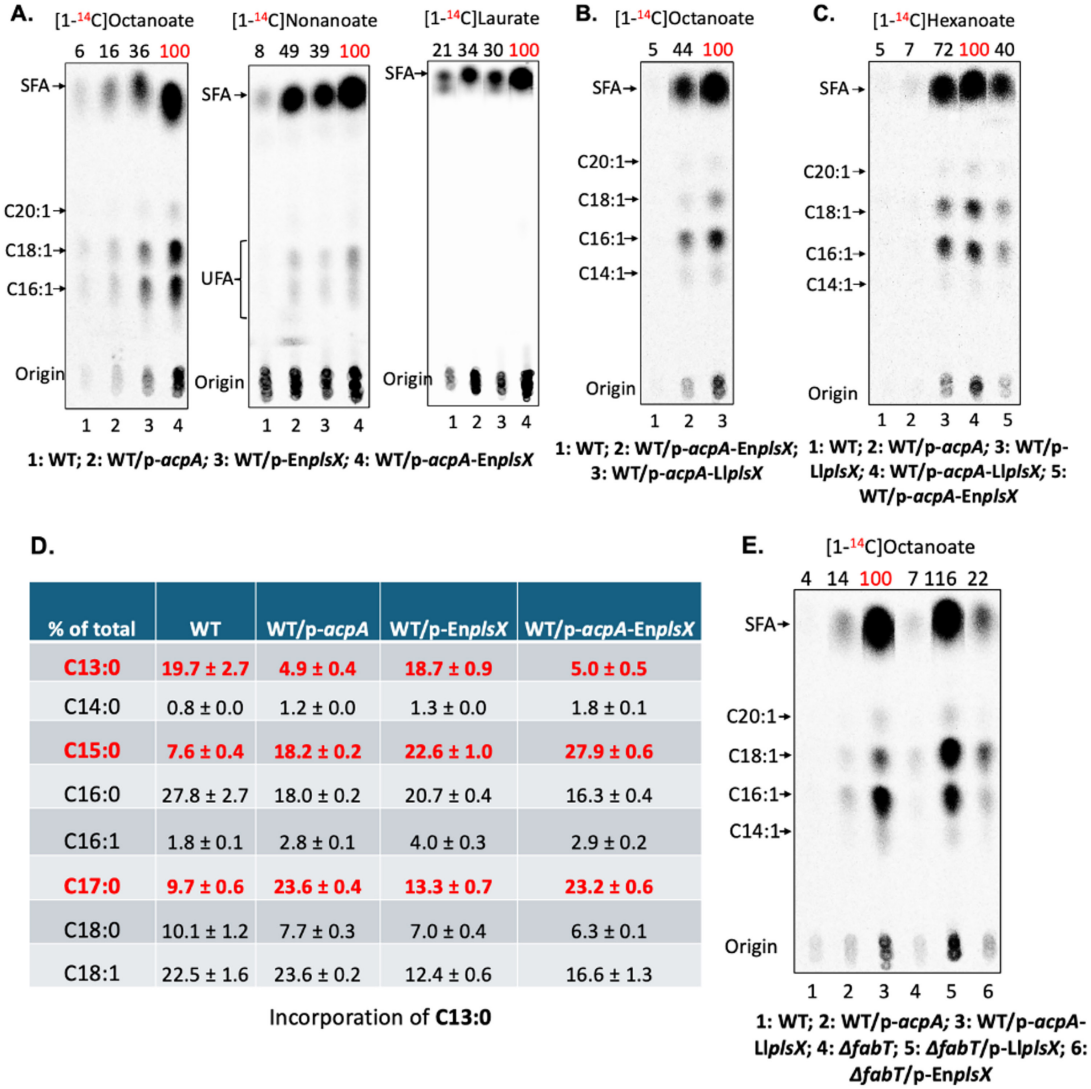
Incorporation and elongation of short‐chain fatty acids relies on coordination between FAS II and PlsX in *E. faecalis*. (A) Incorporation of [^14^C]octanoate (left plate), [^14^C]nonanoate (middle plate), or [^14^C]laurate (right plate) by the *E. faecalis* wild‐type strain overexpressing both AcpA and native PlsX. The numbers above the lanes are the radioactive label incorporations relative to the value (100) for the strain overexpressing AcpA and PlsX. (B) Incorporation of [^14^C]octanoate by the *E. faecalis* wild‐type strain overexpressing AcpA plus either *L. lactis* PlsX expression or *E. faecalis* PlsX overexpression. The numbers above the lanes are the radioactive label incorporations relative to the value (100) for the wild‐type strain overexpressing AcpA coupled with *L. lactis* PlsX expression. (C) Incorporation of [^14^C]hexanoate by the *E. faecalis* wild‐type strain overexpressing AcpA plus either *L. lactis* PlsX expression or *E. faecalis* PlsX overexpression. The numbers above the lanes are the radioactive label incorporations relative to the value (100) for the wild‐type strain overexpressing AcpA coupled to *L. lactis* PlsX expression. (D) GC‐MS analysis for incorporation and elongation of tridecanoic acid (C13:0) in *E. faecalis* wild‐type strain overexpressing AcpA and native PlsX. (E). Incorporation of [^14^C]octanoate by the *E. faecalis ∆fabT* strain expressing *L. lactis* PlsX or overexpressing *E. faecalis* PlsX. The numbers above the lanes are the radioactive label incorporations relative to the value (100) for the wild‐type strain overexpressing AcpA coupled with *L. lactis* PlsX expression. In Panels A–C and E the nonradioactive octanoate and hexanoate were supplied at 1 mM.

Coordination of AcpA and PlsX in incorporation of exogenous fatty acids was assayed using radiolabeled fatty acids. Strains overproducing both proteins incorporated more radiolabeled fatty acids than overexpressing either protein alone (Figure 4A). Substitution of *L. lactis* PlsX for overexpression of the cognate PlsX resulted in further increases in the incorporation of [1‐^14^C]octanoic acid or [1‐^14^C]hexanoic acid (Figure 4B,C). The increased incorporation of heptanoic acid given by *L. lactis* PlsX was confirmed by GC‐MS analysis (Figure S5A). High levels of AcpA and PlsX were also beneficial to the elongation of tridecanoic acid (C13:0) in the *E. faecalis* wild‐type strain (Figure 4D). Expression of *L. lactis* PlsX in the ∆*fabT* strain lacking repression of the *fab* gene operons increased incorporation of [1‐^14^C]octanoic acid, [1‐^14^C]heptanoic acid, and [1‐^14^C]nonanoic acid (Figure 4E; Figure S5B,C).

### 2.5 The Roles of AcpA and AcpB in Incorporation of Short‐Chain Acids Into Phospholipid Acyl Chains

**FIGURE 5:**
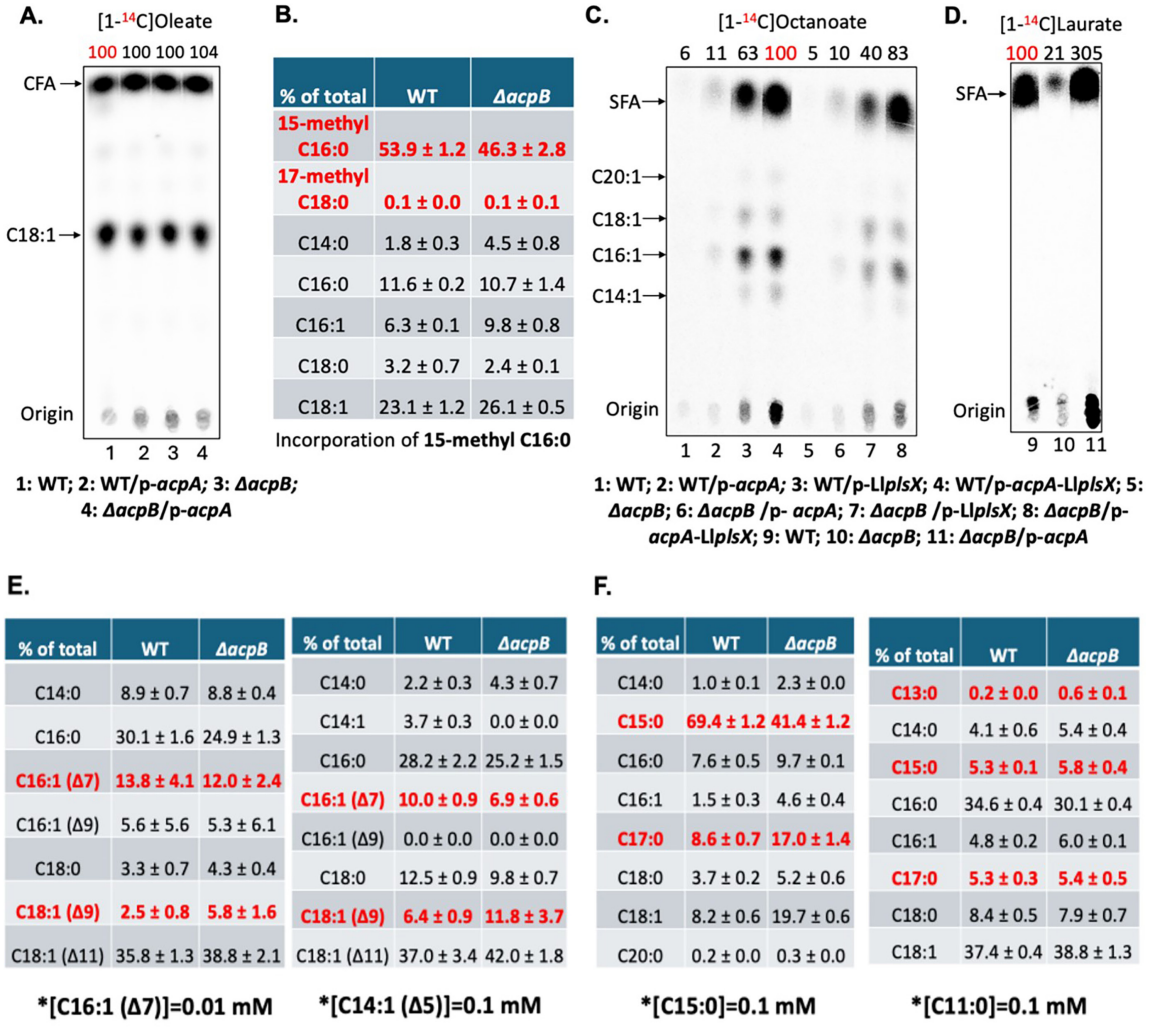
Analysis of the role of the two ACPs of *E. faecalis* in exogenous fatty acid incorporation. (A) Incorporation of [^14^C]oleate by *E. faecalis* wild‐type and *∆acpB* strain overexpressing AcpA. The numbers above the lanes are the radioactive label incorporation values relative to the value (100) for the wild‐type strain. (B) GC‐MS analysis for *E. faecalis* wild‐type and *∆acpB* strains incorporating 15‐methyl palmitic acid (C16:0). (C) Incorporation of [^14^C]octanoate by *E. faecalis* wild‐type and *∆acpB* strain overexpressing AcpA or expressing *L. lactis* PlsX. The numbers above the lanes are the radioactive label incorporation values relative to the value (100) for the wild‐type strain overexpressing AcpA coupled with *L. lactis* PlsX expression. (D) Incorporation of [^14^C]laurate by *E. faecalis* wild‐type and *∆acpB* strains. The numbers above the lanes are the radioactive label incorporation values relative to the value (100) for the wild‐type strain. (E) GC‐MS analysis for *E. faecalis* wild‐type and *∆acpB* strains following incorporation and elongation of exogenous *cis*‐7‐hexadecenoic acid (C16:1) (on left) or *cis*‐5‐tetradecenoic acid (on right). (F) GC‐MS analysis for *E. faecalis* wild‐type and *∆acpB* strains following incorporation and elongation of exogenous pentadecanoic acid (C15:0) (on left) or undecanoic acid (C11:0) (on right). In Panel C, nonradioactive octanoate was supplied at 1 mM.

Prior work had shown that AcpB plays an important role in incorporation of long‐chain fatty acids (Zhu et al. 2019) but its role in incorporation of short‐ and medium‐chain acids was untested. However, long‐chain fatty acid incorporation could also be mediated by AcpA, which is not surprising since although *L. lactis* lacks AcpB, the bacterium efficiently incorporates exogenous fatty acids into phospholipids (Lai and Cronan 2003; Morgan‐Kiss and Cronan 2008). The role of *E. faecalis* AcpA in incorporation is shown by similar [1‐^14^C]oleate incorporation by *∆acpB* strains (Figure 5A) (Dong and Cronan 2022a). Loss of AcpB also had little effect on 15‐methyl palmitic acid incorporation into phospholipid (Figure 5B) but modestly decreased octanoate (C8) incorporation (Figure 5C). In contrast, incorporation of [1‐^14^C]dodecanoic (lauric) acid was strongly dependent on AcpB (Figure 5D). Note that although incorporation of only traces of octanoate precluded comparison of wild‐type and *∆acpB* strains (Figure 5C), substantial incorporation of [^14^C]octanoate was seen when *L. lactis* PlsX was expressed in both strains. The *∆acpB* strain incorporated about one‐third less [^14^C]octanoate than the wild‐type strain (Figure 5C). Overexpression of AcpA in strains expressing *L. lactis* PlsX increased incorporation of [^14^C]octanoate although lack of AcpB still had a weak effect (Figure 5C). Unlike the wild‐type strain (Figure 4A), incorporation of [1‐^14^C]lauric acid was increased by AcpA overexpression in the *∆acpB* strain (Figure 5D), further showing that AcpA plays a role in incorporation of exogenous fatty acids. In contrast incorporation of [1‐^14^C]oleate was unaffected by loss of AcpB or overexpression of AcpA (Figure 5A) since this acid does not require elongation for incorporation into phospholipid.

GC‐MS analysis in triplicate was used to determine the phospholipid acyl chain compositions of the wild‐type strain and *∆acpB* strain supplemented with various unsaturated or saturated fatty acids. Loss of AcpB had little effect on the incorporation of the unsaturated acids, *cis*‐7‐hexadecenoic acid and *cis*‐5‐tetradecenoic acid, and both fatty acids were efficiently elongated (Figure 5E). However, a lack of AcpB resulted in decreased incorporation of pentadecanoic acid into phospholipid although efficient elongation was observed (Figure 5F). Deletion of *acpB* failed to affect the incorporation and elongation of undecanoic acid (Figure 5F). Neither the wild‐type nor *∆acpB* strains were able to grow with the saturated medium‐chain fatty acids (C12:0, C13:0, C14:0, or C15:0) or the unsaturated *cis*‐5‐tetradecenoic acid (Figure S6). Surprisingly, the *E. faecalis ∆fabT* strain showed resistance to inhibition by these saturated fatty acids above (Figure S6).

Note that the reversibility of the PlsX acyltransferase means that the acyl chains attached to AcpB are not necessarily excluded from elongation because acyl‐AcpB chains can be converted to acyl‐AcpA species. This is because the acyl chains of acyl‐AcpB species could be transferred back to phosphate and then transferred to AcpA. Indeed, given such reshuffling of ACP moieties, acyl‐AcpB species could act as a reservoir of acyl chains for transfer to AcpA.

### 2.6 Overexpression of AcpA Allows Elongation of Medium‐Chain Unsaturated Fatty Acids to Phospholipid Acyl Chains

**FIGURE 6:**
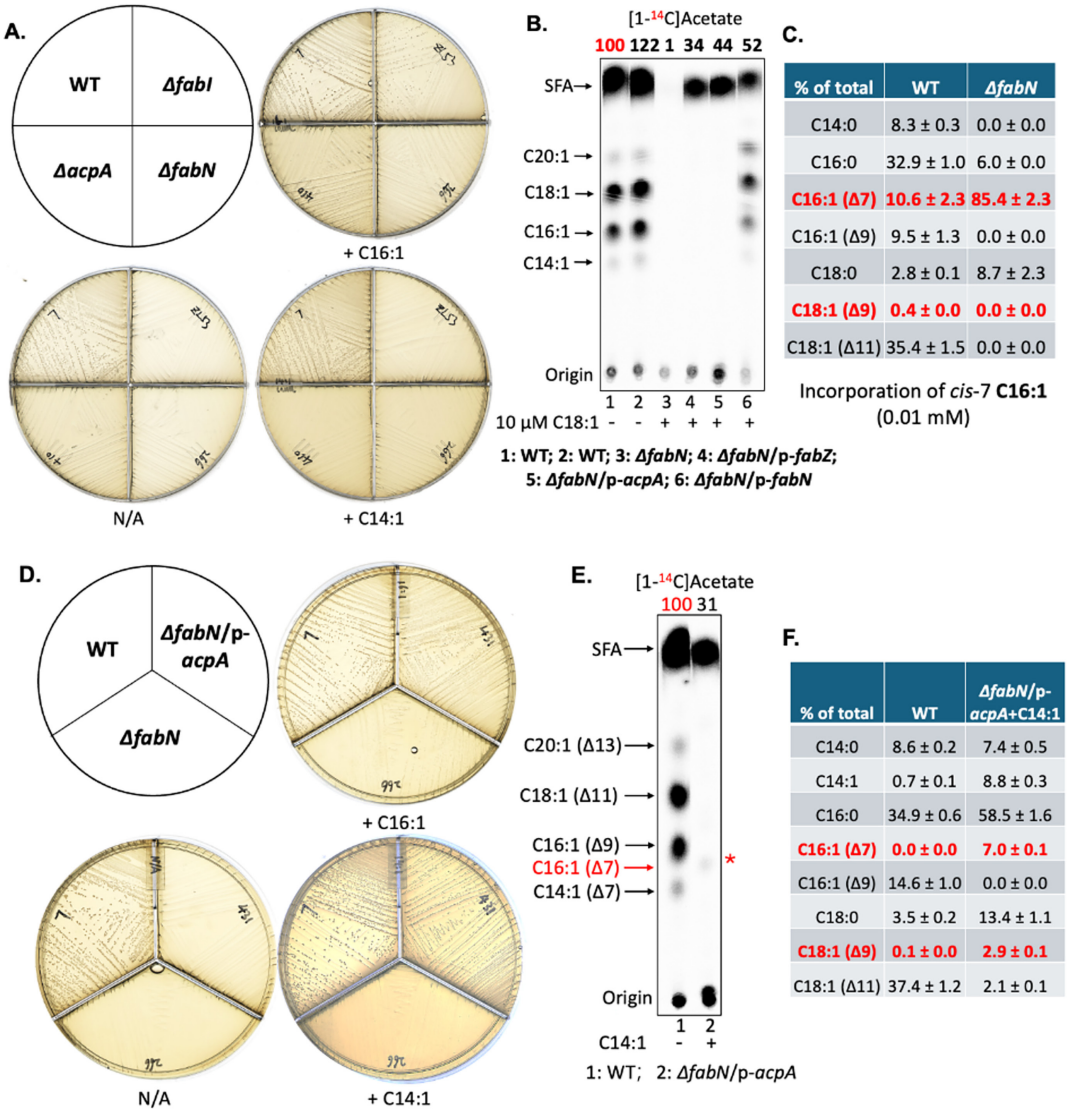
Growth of the *E. faecalis ∆fabN* unsaturated fatty acid auxotrophic strain was supported by *cis*‐5‐tetradecenoic acid upon overexpression of AcpA. (A) Growth of *E. faecalis* fatty acid synthesis gene deletion strains in the presence of *cis*‐5‐tetradecenoic acid (C14:1) or *cis*‐7‐hexadecenoic acid (C16:1). (B) *De novo* synthesis of phospholipid fatty acyl chains in a *E. faecalis ∆fabN* strain overexpressing AcpA in the presence of 10 μM *cis*‐vaccenic acid (C18:1 ∆11) lane 2 . The numbers above the lanes are the radioactive label incorporations relative to the value (100) for the wild‐type strain cultured without exogenous fatty acids. (C) GC‐MS analysis for *E. faecalis ∆fabN* strain incorporating *cis*‐7‐hexadecenoic acid (C16:1). (D) Growth of the *E. faecalis ∆fabN* strain overexpressing AcpA in the presence of *cis*‐5‐tetradecenoic acid (C14:1). (E) *De novo* synthesis of phospholipid fatty acyl chains by *E. faecalis ∆fabN* strain overexpressing AcpA in the presence of *cis*‐5‐tetradecenoic acid (C14:1). The numbers above the lanes are the radioactive label incorporations relative to the value (100) for the wild‐type strain cultured without exogenous fatty acids. The red star “*” denotes the *cis*‐7 C16:1 synthesized by elongation of *cis*‐5 C14:1 by the *∆fabN* strain overexpressing AcpA in lane 2. (F) GC‐MS analysis for incorporation and elongation of *cis*‐5‐tetradecenoic acid (C14:1) in *E. faecalis ∆fabN* strain overexpressing AcpA. In Panels B, E, and F, the strains were first cultured with 0.01 mM *cis*‐11‐vaccenic acid (C18:1) overnight and then transferred to the new medium at an OD_600_ of 0.1. In Panel C, the strains were first cultured with 0.01 mM *cis*‐7‐hexadecenoic acid (C16:1) overnight and then transferred to the same new medium at an OD_600_ of 0.1. In Panels E and F, the *cis*‐5‐tetradecenoic acid was supplied at 0.01 mM.

**FIGURE 7:**
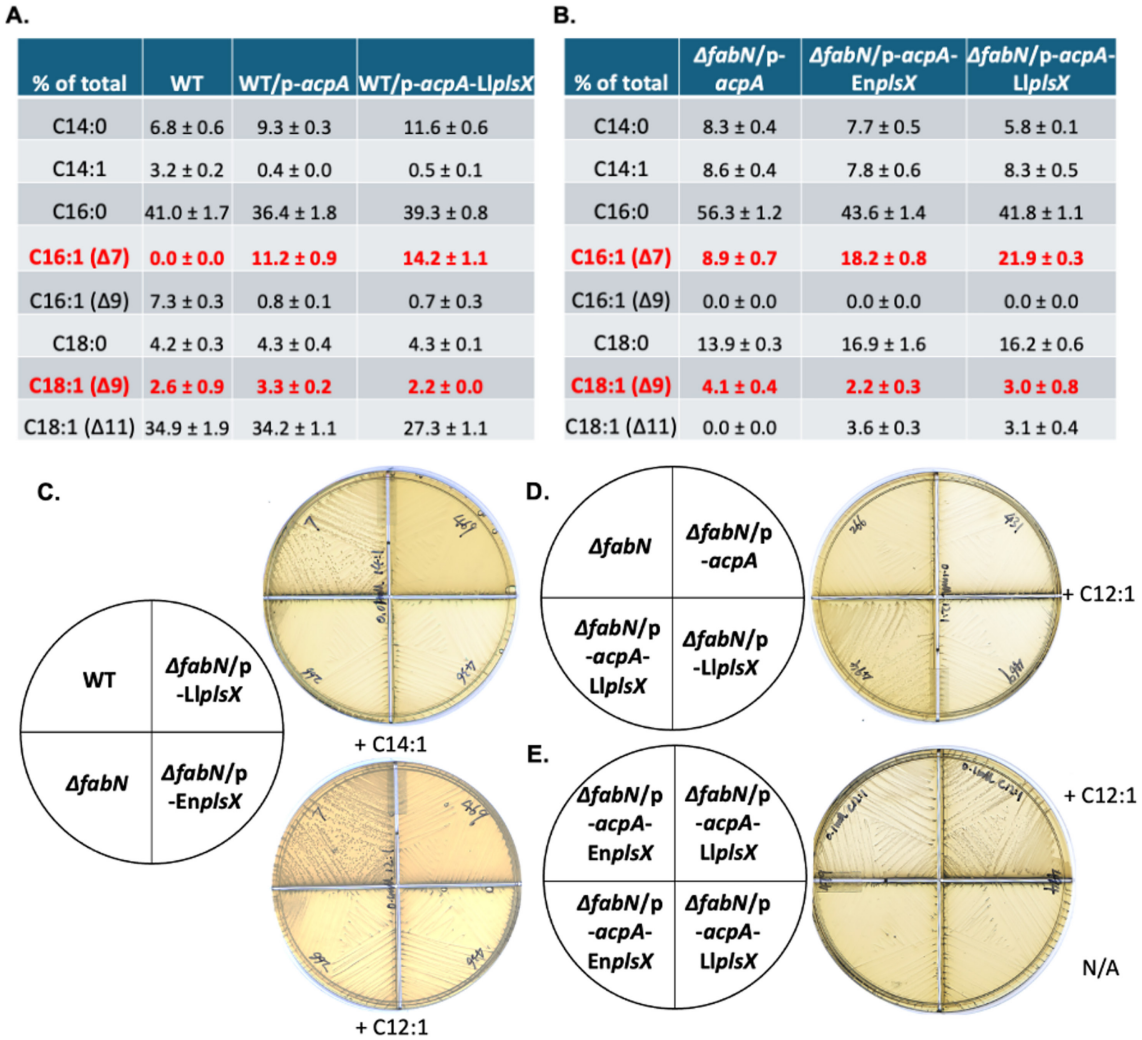
Growth of the *E. faecalis ∆fabN* unsaturated fatty acid auxotrophic strain was supported by *cis*‐5‐dodecenoic acid. (A and B) GC‐MS analysis for incorporation and elongation of *cis*‐5‐tetradecenoic acid by *E. faecalis* wild‐type strain (A) or *∆fabN* strain (B) with overexpression of AcpA and PlsX. (C) Growth of the *E. faecalis ∆fabN* strain overexpressing PlsX in the presence of *cis*‐5‐tetradecenoic acid (C14:1) (top plate) or *cis*‐5‐dodecenoic acid (C12:1) (bottom plate). (D and E). Growth of the *E. faecalis ∆fabN* strain overexpressing AcpA plus either *L. lactis* PlsX expression (D) or *E. faecalis* PlsX overexpression (E) in the presence of *cis*‐5‐dodecenoic acid (C12:1). In Panel B, the *∆fabN*‐related strains were first cultured with 0.01 mM *cis*‐11‐vaccenic acid (C18:1) overnight and then transferred to the medium with 0.01 mM *cis*‐5‐tetradecenoic acid (C14:1) at an OD_600_ of 0.1.

Given the increased incorporation of short‐chain acids seen upon overproduction of AcpA and/or PlsX, we turned to our prior unsuccessful attempts to support the growth of *∆fabN* unsaturated fatty acid auxotrophs by putative short unsaturated pathway intermediates. The protein encoded by *fabN* functionally replaces the *E. coli fabA* gene encoding the 3‐hydroxydecanoyl‐ACP dehydratase/isomerase that inserts the double bond, (Wang and Cronan 2004; Dong and Cronan 2022b; Diederich et al. 2016). Hence, *cis‐*3‐decenoyl‐ACP produced by FabN should be elongated to *cis*‐5‐dodecenoyl‐ACP, then to *cis*‐7‐tetradecenoyl‐ACP and finally to the *cis*‐9‐hexadecenoate and *cis*‐11‐octadecenoate species found in the *E. faecalis* phospholipids. Therefore, we were puzzled by our inability to replace the oleate used to support the growth of the *∆fabN* strain with C14 and C12 unsaturated acids (Figures 6 and 7).

As first seen with the medium‐chain saturated acids, overexpression of AcpA allowed the growth of the *∆fabN* strain with *cis*‐5‐tetradecenoic acid (the *cis‐*7‐isomer was not available) (Figure 6D) and resulted in the appearance of phospholipid *cis*‐7‐hexadecenote and oleate (*cis*‐9‐octadecenote) acyl chains (Figure 6F). We also found that overexpression of AcpA gave increased *cis*‐7‐hexadecenote and oleate acyl chains in the phospholipids of the *E. faecalis* wild‐type strain (Figure 7A). High levels of either of the native or *L. lactis* PlsX proteins were ineffective although these were beneficial when expressed together with AcpA overproduction in the *∆fabN* strain as increased contents of phospholipid *cis*‐7‐hexadecenote and oleate acyl chains were seen (Figure 7B,C).

Note that *cis*‐7‐hexadecenoic acid (C16:1) could weakly restore growth of not only the *∆fabN* strain but also of *∆fabI* and *∆acpA* fatty acid auxotrophs (Figure 6A) (Zhu et al. 2013; Dong and Cronan 2022a). However, only low concentrations could be tested due to toxicity (see below). Further examination of phospholipid fatty acyl chain synthesis by [1‐^14^C]acetate labeling showed the failure to synthesize saturated fatty acids by *E. faecalis ∆fabN* strain, which could be restored by overexpression of AcpA (Figure 6B). The *∆fabN* strain failed to elongate *cis*‐7‐hexadecenoic acid to produce oleoyl phospholipid chains (Figure 6C). Note that expression of *fab* genes in the *E. faecalis ∆fabN* strain was repressed relative to the wild‐type strain (Figure S7). The presence of *L. lactis* PlsX had little or no effect on incorporation of *cis*‐5‐tetradecenoic acid in the *E. faecalis* wild‐type strain when AcpA was overexpressed (Figure 7A). In contrast, the shorter *cis*‐5‐dodecenoic acid was an effective supplement only when both AcpA and *L. lactis* PlsX were expressed at high levels (Figure 7DE). Use of the unnatural *cis*‐5‐tetradecenoic acid had the advantage that the double bond positions of the elongation products differed from those of the wild‐type unsaturated acyl chains and hence their incorporation into phospholipids could be detected by GC–MS. This is not the case for the *cis*‐5‐dodecenoic acid elongation products because they are those normally present in *E. faecalis* phospholipids. Note that for both *cis*‐7‐hexadecenoic acid and *cis*‐9‐hexadecenoic acid (palmitoleic acid) concentrations greater than 0.02 mM were growth inhibitory (Figure S8). This has been observed by others (Rashid et al. 2023). The reason for toxicity of hexadecenoic acids in *E. faecalis* is unknown. However, palmitoleic acid has been reported to be a potent inhibitor of *Staphylococcus aureus* growth due to membrane depolarization whereas oleic acid (*cis*‐9‐octadecenoic acid) was nontoxic (Parsons et al. 2012).

## 3 Discussion

The utilization of short‐ and medium‐chain fatty acids by *E. faecalis* is complex. Several different players and many different fatty acid species that require (or do not require) increased expression of AcpA and or a PlsX. Table 1 is an attempt to illustrate the complexity. Short‐chain fatty acids were efficiently elongated by *E. faecalis* only when PlsX was highly expressed and in this process *L. lactis* PlsX was superior to *E. faecalis*. For example, expression of *L. lactis* PlsX allowed efficient utilization of octanoic acid (C8) (Figure 3C) whereas overexpression of the *E. faecalis* PlsX was less effective. However, the two PlsX proteins were similarly effective with the C18 acids, oleate and stearate (Figure 3AB). The increased activity of *L. lactis* PlsX towards short and medium acyl chains seems likely to reflect the growth environments of dairy industry *L. lactis* strains. Note that enterococci including *E. faecalis* are found in some dairy products. An example is kumis, a regional Colombian traditional fermented milk that contains several enterococcal strains (Chaves‐Lopez et al. 2011). *L. lactis* also dwells on plants where it utilizes complex plant polymers for growth. Domestication of a plant *L. lactis* strain to milk was examined after 1000 generations in milk (Bachmann et al. 2012). During domestication the genome of the plant strain underwent a series of changes in nitrogen metabolism pathways together with losses in the plant polymer utilization pathways.

**TABLE 1:** Summary of effects of AcpA, AcpB and PlsX species on incorporation of exogenous fatty acids by the *E. faecalis* wild‐type strain.

Exogenous fatty acids	No overexpression	AcpA overexpression	*E. faecalis* PlsX overexpression	*L. lactis* PlsX overexpression	Loss of AcpB
C6:0^a^	(+)	++	ND	++++	ND
C7:0	(+)	ND	ND	++++	ND
C8:0^b^	(+)	++	+++	++++	(+)
C9:0	(+)	++	++	++++	ND
C11:0	+	++	ND	++++	+
C12:0	++	+++	+++	ND	+
C13:0	+++	+++	++++	ND	ND
C14:1 (∆5)^c^	+	+++	ND	ND	ND
C15:0	++++	++++	ND	++++	+++
C16:1 (∆7)^d^	++	ND	ND	ND	++
15‐Methyl C16:0	+++	ND	ND	ND	+++

*Note:* The number of plus signs represents the capacity of each protein in promoting incorporation of each fatty acid species. Values in parentheses indicate < 10% incorporation relative to the maximal incorporation of that chain. ND denotes no data. The red plus signs determinations are based on thin layer chromatography analysis of cells following incorporation of [^14^C]fatty acids whereas the determinations for the blue plus signs are based on gas chromatography–mass spectrometry analyses of incorporated unlabeled fatty acids.

^a^ This result was obtained at 1 mM C6:0 (Figure 4C).

^b^ This result was obtained at 1 mM C8:0 (Figures 4A,B and 5C).

^c^ This result was obtained at 0.01 mM C14:1 (∆5) (Figure 7A) whereas 0.1 mM C14:1 (∆5) gave significantly greater incorporation by the wild‐type strain (Figure 5E).

^d^ This result was obtained at 0.01 mM C16:1 (∆7) (Figures 5E and 6C; Figure S4B).

In industrial processes *L. lactis* strains are used to make cheeses and other dairy products from bovine milk which contains abundant triglycerides having high contents of short and medium length acyl chains. Milk contains a lipase that releases the triglyceride acyl chains but this is often intentionally heat inactivated and replaced with a designated lipase to provide release of specific fatty acids to impart desired flavors (Lubary et al. 2009). Sheep or goat milk, which are higher in short‐chain fatty acids is often treated with a specific lipase to make cheese having a sour taste due to released fatty acids released (e.g., feta) (Omar et al. 2016). Hence, in the dairy industry *L. lactis* lives in an environment that contains very high concentrations of the short‐ and medium‐chain fatty acids. A reasonable tactic for such *L. lactis* strains would be to utilize these short chains to make phospholipid acyl chains and thereby save a significant amount of the energy required for complete *de novo* synthesis. Indeed, prior work from this laboratory showed that deuterated octanoic acid could be elongated to phospholipid acyl chains by *L. lactis* (Morgan‐Kiss and Cronan 2008). Since *L. lactis* and *E. faecalis* are both Lactobacillales, it is not surprising that their fatty acid/phospholipid synthesis pathways are similar. Indeed, a dairy *L. lactis* can be considered a stripped‐down version of *E. faecalis*. The *L. lactis subsp. lactis* Il1403 genome is ~30% smaller than that of *E. faecalis*, a reflection the metabolic simplification characteristic of the evolution of lactic acid bacteria (Makarova et al. 2006). However, *L. lactis* lacks AcpB and the genome neighborhoods of *fabT* and *plsX* differ in the two bacteria. *L. lactis* also lacks *fabK*, a cryptic *E. faecalis* gene (Bi et al. 2014). The requirement for elevated PlsX expression for efficient utilization and elongation of short‐chain acids argues that *E. faecalis* PlsX expression might be upregulated in the mammalian gastrointestinal tract to take advantage of environmental short‐ and medium‐chain acids. This would be in contrast with the laboratory setting where PlsX expression appears constitutive (Zhu et al. 2019). The levels of these fatty acids in the human gastrointestinal tract are indeterminant because the levels would depend on the diets of the subjects as reported for serum fatty acid levels (Zeleniuch‐Jacquotte et al. 2000). Another unknown factor is competition with other members of the microbiome. Hence, this is in direct contrast to dairy *L. lactis* strains where the environmental fatty acids can be predicted.

Increases in AcpA expression had minimal effect on elongation of short‐chain acids but was required for elongation of the medium‐chain unsaturated acid, C14∆5, to chain lengths suitable for incorporation into phospholipids and thereby allowing growth of the *∆fabN* deletion strain whereas the shorter C12∆5 acid required overproduction of both AcpA and high‐level PlsX expression (Figure 6D; Figure 7DE). This argues that the C14∆5 acid was an efficient PlsX substrate whereas the C12∆5 acid was a significantly poorer substrate.

As noted above the regulation of *de novo* fatty acid synthesis in *E. faecalis* and related bacteria is exerted by the FabT repressor complexed with an acyl‐ACP (either AcpA or AcpB) carrying a long acyl chain (≥ C12) (Lambert et al. 2023, 2022; Zou et al. 2022). This regulation is nicely illustrated by a recent study of the effects of loss of the MprF1and MprF2 proteins required for resistance to cationic antimicrobial peptides (Rashid et al. 2017). Loss of these resistance proteins resulted in dramatic decreases in the synthesis of cationic phospholipid species. This also resulted in decreased *de novo* fatty acid synthesis and accumulation of long‐chain acyl‐AcpA species. A straightforward explanation of these results is that the accumulated acyl‐AcpA species bind FabT and shut down transcription of the fatty acid synthesis genes. In this scenario, an ∆*mprF2 ∆mprF2* ∆*fabT* triple deletion strain should have increased acyl‐AcpA levels relative to the ∆*mprF2 ∆mprF2* strain.

In contrast to unsaturated medium‐chain acid incorporation, incorporation of saturated medium chains proceeds efficiently without the need for high levels of PlsX (Figures 3G and 4D; Figure 5B,D,F and Table 1). Odd chain length and methylated acids were used to allow the incorporation/elongation products to be distinguished from the normal acyl chains by GC–MS. The most straightforward explanation for the complex pattern of utilization of short‐ and medium‐chain fatty acids by the *E. faecalis* fatty acid synthesis pathway (Table 1) is the FakB component of the FakAB system. Although *E. faecalis* encodes four such fatty acid‐binding proteins, the rationale for multiple FakBs is unclear. This is unlike the case in *Streptococcus pneumoniae* where the three FakBs have distinct fatty acid preferences (Gullett et al. 2019). FakB1 selectively binds the saturated fatty acid palmitate, whereas FakB2 preferred the unsaturated fatty acid, oleate, and FakB3 preferred the diunsaturated acid, linoleate. We have repeatedly been unable to detect such selective fatty acid utilization by the four *E. faecalis* FakB proteins (Zou et al. 2022) (Figure 2; Figure S3), although efforts are continuing. However, it should be noted that in addition to FakB binding, a given exogeneous fatty acid must pass two other filters of unknown specificity. One is the membrane bound acyl transferases, PlsY and PlsC that attach the acyl chains to glycerol‐3‐phosphate in synthesis of phosphatidic acid, the key phospholipid synthetic intermediate. Another filter for some chain lengths is transfer from the Fak fatty acid kinase system to AcpB (Table 1). Note that any chains that are elongated but fail to achieve the length required for phospholipid synthesis would not be observed in our experiments.

We assume that once an acyl‐AcpA thioester enters the fatty acid synthesis cycle, this is irreversible since the malonyl building block is decarboxylated. However, it is unknown how efficiently a given acyl‐AcpA species derived from an exogenous acyl chain competes with *de novo* synthesized acyl‐AcpA species for elongation. Another irreversible reaction is phosphatidic acid synthesis which normally proceeds with long chains. However, this is not absolute. Prior work in which the only *E. faecalis* ACP present was a multiply mutant AcpB derivative intended to mimic AcpA gave an atypical phospholipid acyl chain composition (Dong and Cronan 2022a). Most of the phospholipid acyl chains were C12 and C14 species which are very minor or absent components of wild‐type *E. faecalis* phospholipids (Dong and Cronan 2022a). A similar shift to short phospholipid chains in conditions deficient in fatty acid synthesis was reported in *E. coli* (Harder et al. 1974).

## 4 Materials and Methods

### 4.1 Materials

The 15‐methyl palmitic acid and *cis*‐5‐tetradecenoic acid were purchased from Cayman Chemicals and all the other fatty acids, hexanoyl‐CoA, octanoyl‐CoA, and antibiotics were purchased from Sigma‐Aldrich. The media were purchased from Fisher Scientific. The DNA polymerase, restriction endonuclease, T4 ligase and Gibson Assembly Cloning Kit were purchased from New England Biolabs. Sodium[1‐^14^C]acetate (specific activity, 57.0 mCi/mmol), sodium[1‐^14^C]octanoate (specific activity, 57.0 mCi/mmol), and [1‐^14^C]stearic acid (specific activity, 53.0 mCi/mmol) were provided by Moravek, Inc. and the [2‐^14^C]malonyl coenzyme A (specific activity, 55.0 mCi/mmol), sodium[1‐^14^C]hexanoate (specific activity, 55.0 mCi/mmol), [1‐^14^C]nonanoic acid (specific activity, 53.0 mCi/mmol), and [1‐^14^C]lauric acid (specific activity, 55.0 mCi/mmol) were purchased from American Radiolabeled Chemicals. Ni‐NTA resin was from Invitrogen and the DNA purification kits were from Qiagen. Silver nitrate silica gel thin layer plates were purchased from Analtech and M17 Broth was from Becton Dickenson. All the other reagents were of the highest available quality. Oligonucleotide primers were synthesized by Integrated DNA Technologies and DNA sequencing was performed by ACGT, Inc.

### 4.2 Bacterial Strains, Plasmids, and Incubations

The bacterial strains and plasmids used in this study are listed in Table S1 and the primers used for this study are listed in Table S2. *E. coli* cultures were incubated at 37°C in Luria‐Bertani medium (tryptone, 10 g/L; yeast extract, 5 g/L; NaCl, 10 g/L) whereas *E. faecalis* cultures were grown at 37°C in M17 medium (BD Difco). Antibiotics were added at the following concentrations (in mg/L): sodium ampicillin, 100 for *E. coli*; kanamycin sulfate 50 for *E. coli*; chloramphenicol 30 for *E. coli*; erythromycin at 250 for *E. coli* and 5 for *E. faecalis*. Hexanoic acid, heptanoic acid, octanoic acid, and nonanoic acid were added at 1 or 0.1 mM whereas *cis*‐5‐tetradecenoic acid and *cis*‐vaccenic acid were added at 0.01 or 0.1 mM. The *cis*‐7‐hexadecenoic acid was added at 0.01 or 0.02 mM. The other fatty acids were added at 0.1 mM unless otherwise stipulated.

### 4.3 *E. faecalis* MCAT Assay

The expression plasmid for *E. faecalis* malonyl‐CoA‐ACP transacylase (FabD) was constructed by inserting the *fabD* coding fragment amplified by EffabD NdeI F and EffabD EcoRI R into pET28b vector at the NdeI and EcoRI sites. To express *E. faecalis* FabD, *E. coli* Rosetta cells transformed with the expression plasmid above were incubated at 37°C to OD_600_ of 0.6 and then induced with 1 mM isopropyl β‐D‐1‐thiogalactopyranoside (IPTG) for 4 h. The cells were harvested, washed with phosphate‐buffered saline (PBS), resuspended with lysis buffer containing 50 mM sodium phosphate (pH 8.0), 0.3 M NaCl, 10 mM imidazole, and 1 mM DTT and then lysed with a French Pressure Cell. The supernatant after centrifugation was loaded onto Ni‐NTA column and the FabD protein was eluted with 0.25 M imidazole, dialyzed, and stored with 20% glycerol at −80°C. To characterize the biochemical function of purified *E. faecalis* FabD in vitro, *E. faecalis* holo‐ACPs were prepared as demonstrated in the previous work (Zhu et al. 2019; Zou, Dong, and Cronan 2023; Zou et al. 2022) and then mixed with purified *E. faecalis* FabD protein and 2 μCi/mL [2‐^14^C]malonyl‐coenzyme A in buffer containing 50 mM Tris–HCl (pH 8.0) and 1 mM DTT. The reaction was processed at 37°C for 2 h and the products were analyzed by conformation‐sensitive 1 M urea‐20% polyacrylamide gel electrophoresis.

### 4.4 Synthesis of Short‐Chain Fatty Acyl‐ACPs

The synthesis of short‐chain fatty acyl‐ACPs was through fatty acid kinase (Fak)‐phosphate acyltransferase (PlsX) assay described in the previous work (Zou et al. 2022). The *E. coli* ACP (apo/holo), *E. faecalis* AcpA, *E. faecalis* AcpB, *E. faecalis* FakA, *E. faecalis* FakB1, FakB2, FakB3, and FakB4, *E. faecalis* PlsX and *L. lactis* PlsX were from lab stock and their purification assay were discussed previously (Zhu et al. 2013, 2019; Zou et al. 2022). Briefly, holo‐ACP species were mixed with various 0.2 mM short‐chain fatty acids respectively in the presence of *E. faecalis* 1 μM FakA, 1 μM FakB, and 0.5 μM PlsX proteins in buffer containing 50 mM Tris–HCl (pH 8.0), 2 mM MgCl_2_, 1 mM DTT and 0.2 mM ATP and then incubated at 37°C for 1 h. The products were analyzed on 2 M urea‐18% PAGE conformation‐sensitive gel electrophoresis. The standard acyl‐ACPs were synthesized from acyl‐CoAs by *Bacillus subtilis* 4′‐phosphopantetheinyl transferase Sfp or free fatty acids by *Vibrio harveyi* acyl‐ACP synthetase AasS as described previously (Jiang, Chan, and Cronan 2006; Zhu et al. 2019; Zou et al. 2022).

### 4.5 Construction of *L. lactis plsX*, *E. faecalis* *acpA* and *fabZ* Expression Plasmids

The construction of *L. lactis plsX*‐expression plasmid was modified from the construction of *E. faecalis plsX* expression plasmid described previously (Marelli and Magni 2010; Zou et al. 2022; Zou, Dong, and Cronan 2023). The *L. lactis plsX* gene amplified from the genomic sequence (Bolotin et al. 2001) using primer set LlplsX F1 and LlplsX R1 was ligated with linearized pQZ28 vector amplified using primer set pQZ28 F1 and pQZ28‐p32 R1 through Gibson assembly assay. To construct the *E. faecalis acpA*‐expression plasmid, the *acpA* gene was amplified from genomic DNA using primer set EfacpA *NcoI* F and EfacpA *EcoRI* R and then inserted into the *NcoI* and *EcoRI* sites of vector pQZ28.

The construction of the *E. faecalis fabZ*‐expression plasmid followed essentially the same procedures as the construction of the *E. faecalis acpA* expression plasmid above. The *fabZ* gene was amplified from genomic DNA using primer set EffabZ *NcoI* F and EffabZ *EcoRI* R and then inserted into the *NcoI* and *EcoRI* sites of vector pQZ28 (Marelli and Magni 2010) as described above.

### 4.6 Construction of Co‐Expression Plasmids of *E. faecalis acpA* and *E. faecalis* or *L. lactis plsX*

To construct the plasmid that co‐expressed *E. faecalis acpA* and *E. faecalis plsX*, the *E. faecalis acpA* gene together with upstream p32 promoter was amplified from the constructed *acpA*‐expression plasmid above using primer set p32 F2 and EfacpA R2 while the *E. faecalis plsX* gene together with upstream p32 promoter was amplified from *E. faecalis plsX*‐expression plasmid constructed previously using primer set p32 F3 and EfplsX R (Zou, Dong, and Cronan 2023). The two fragments were ligated with the linearized pQZ28 vector amplified with primer set pQZ28 F2 and pQZ28 R2 through Gibson assembly.

The construction of the co‐expression plasmid of *E. faecalis acpA* and *L. lactis plsX* was essential as the methods described above. In this case, the *E. faecalis acpA* gene together with upstream p32 promoter was amplified using primer set p32 F2 and EfacpA R2 whereas the *L. lactis plsX* gene together with upstream p32 promoter was amplified from *L. lactis plsX*‐expression plasmid above using primer set p32 F3 and LlplsX R2. The linearized pQZ28 vector was amplified by primer set pQZ28 F3 and pQZ28 R2.

### 4.7 Thin Layer Chromatography (TLC) Analysis of Radioactive Labeled Fatty Acid Methyl Esters From Bacterial Phospholipids

To test phospholipid fatty acyl chain synthesis, 5 mL *E. faecalis* cultures were inoculated at OD_600_ of 0.1 in M17 medium and incubated at 37°C for 6 h (early‐ to mid‐exponential phase) in the presence of 1 μCi/mL sodium [1‐^14^C]acetate with or without a single exogenous fatty acid at the concentration described above. The cultures were normalized by OD_600_ and the collected cells were lysed with methanol‐chloroform (2:1) solution and the phospholipids were extracted with chloroform and dried under nitrogen. The fatty acyl groups were methylated by 25% (w/v) sodium methoxide, extracted by hexanes, and processed for TLC analysis on Analtech silica gel containing 20% silver nitrate in toluene at −20°C. The TLC plates containing the [^14^C]‐labeled fatty acid methyl esters were exposed and quantitated by phosphorimaging on a GE Typhoon FLA700 Scanner, and the data wwere analyzed by ImageQuant TL software. Note that sodium methoxide catalyzes transesterification but not esterification. Hence all fatty acyl chains must have been in ester linkage (i.e., in complex lipids) to be included in our analyzes.

To test incorporation and elongation of octanoic acid (C8:0), *E. faecalis* strains were inoculated at OD_600_ of 0.1 in 5 mL of M17 medium containing 0.1 μCi/mL sodium [1‐^14^C]octanoate plus 1 or 0.1 mM non‐radioactive octanoic acid and cultured at 37°C for 6 h (early‐ to mid‐exponential phase). The cells were washed with phosphate‐buffered saline and the phospholipids were extracted, methylated, and analyzed as described above. The same assay was also utilized for examine *E. faecalis* strains incorporating and elongating hexanoic acid (C6:0), heptanoic acid (C7:0), nonanoic acid (C9:0), and lauric acid (C12:0).

To test incorporation of long chain saturated fatty acids, *E. faecalis* strains were inoculated at OD_600_ of 0.1 in 5 mL of M17 medium containing 0.1 μCi/mL [1‐^14^C]stearic acid plus 0.1 mM non‐radioactive palmitate, cultured at 37°C for 6 h (early‐ to mid‐exponential phase) and processed for TLC analysis as described above. To test the incorporation of long‐chain unsaturated fatty acids, *E. faecalis* strains were inoculated at OD_600_ of 0.1 in 5 mL of M17 medium containing 0.1 μCi/mL [1‐^14^C]oleic acid plus 0.1 mM non‐radioactive oleate, cultured at 37°C for 6 h (early‐ to mid‐exponential phase) and processed for TLC analysis as described above.

### 4.8 Conversion of Fatty Acids to Acyl‐ACPs by the Fak System

Briefly, holo‐ACP species (about 100 μM) were mixed with various 0.2 mM short‐chain fatty acids respectively in the presence of the *E. faecalis* proteins 1 μM FakA, 1 μM FakB, and 0.5 μM PlsX in buffer containing 50 mM Tris–HCl (pH 8.0), 2 mM MgCl_2_, 1 mM DTT and 2 mM ATP and then incubated at 37°C for 1 h. The products were generally analyzed on 2 M urea‐18% PAGE conformation‐sensitive gel electrophoresis (Cronan and Thomas 2009) in Tris (3 g/L)‐glycine (14.4 g/L) buffer (120 V for 90 min) followed by Coomassie Blue staining. The standard acyl‐ACPs were synthesized from acyl‐CoAs (0.2 mM) using the *Bacillus subtilis* 4′‐phosphopantetheinyl transferase Sfp in the presence of MgCl_2_ (2.5 mM) or from free fatty acids (0.2 mM) by *Vibrio harveyi* Acyl‐ACP synthetase (AasS) in the presence of ATP (2 mM) and MgCl_2_ (2.5 mM) in Tris–HCl (50 mM, pH 8.0) as described previously (Zou et al. 2022).

### 4.9 Gas Chromatography–Mass Spectrometry Analysis of Phospholipid Acyl Chains

To analyze the fatty acyl chains of bacterial phospholipids, *E. faecalis* strains were inoculated at an OD_600_ of 0.1 in M17 medium with or without the presence of a single fatty acid at the concentration given and cultured at 37°C for 6 h. The protocol for the conversion of phospholipids to fatty acid methyl esters was used for TLC analysis above and the extracted products were submitted for gas chromatography–mass spectrometry analysis to the University of Illinois Carver Metabolics Center where the data were collected in triplicate.

### 4.10 β‐Galactosidase Assays

The *lacZ* reporter plasmids expressing *E. coli* β‐galactosidase from promoters of *E. faecalis* fatty acid biosynthesis (*fab*)‐related genes were constructed in the previous work (Zou et al. 2022) Briefly, *E. faecalis* strains transformed with the above *lacZ* reporter plasmids were cultured to mid‐log phase at 37°C and the harvested cells were washed by phosphate‐buffered saline (PBS), resuspended in Z‐buffer, lysed with sodium dodecyl sulfate (SDS) and chloroform, and assayed for β‐galactosidase activity (Bi et al. 2014; Zou, Dong, and Cronan 2023). The data were collected in triplicate.

## Author Contributions

**Qi Zou:** conceptualization, investigation, writing – original draft, methodology, writing – review and editing. **Huijuan Dong:** investigation, methodology, validation, formal analysis. **John E. Cronan:** conceptualization, funding acquisition, writing – original draft, writing – review and editing, methodology, validation.

## Conflicts of Interest

The authors declare no conflicts of interest.

## Supporting information


Data S1.

## Data Availability

The data that support the findings of this study are available from the corresponding author upon reasonable request.
