# Supplementary material for: The Enteric Bacterium Enterococcus faecalis Elongates and Incorporates Exogenous Short and Medium Chain Fatty Acids Into Membrane Lipids
Source: Mol Microbiol. 2024 Oct 8;122(5):757–71. doi: 10.1111/mmi.15322 (PMC11586512; doi:10.1111/mmi.15322)
Supplement: Supplementary file 1 — Data S1. [file MMI-122-757-s001.docx]

Supplement to

***Enterococcus faecalis* incorporates and elongates exogenous fatty acids for synthesizing phospholipids**

Qi Zou, Huijuan Dong and John E. Cronan

**Table S1 Strains and Plasmids**

| **Strains and Plasmids** | **Description** | **Source** |
| --- | --- | --- |
| **Strains** |  |  |
| *E. coli Rosetta* | *ompT hsdSB* (rB^-^ mB^-^) *gal dcm* (DE3) pRARE (Cm^r^) | Novagen |
| *E. faecalis* FA2-2 | Wild Type | Lab Store |
| *L. lactis*  Il1403 | *Lactococcus lactis* *subsp. lactis* Il1403 | (Bolotin et al., 2001) |
| *E. faecalis* ZL116 | *∆fabT* | Zhu et al., 2019 |
| *E. faecalis* QZ157 | *∆plsX* | Zou et al., 2023 |
| *E. faecalis* DHJ523 | *∆acpB* | Zou et al., 2022 |
| *E. faecalis* DHJ484 | *∆acpA* | Dong & Cronan, 2022a |
| *E. faecalis* DHJ497 | *∆fabN* | Dong & Cronan, 2022b |
| *E. faecalis* QZ55 | *∆acpB::cat* | Zou et al., 2022 |
| *E. faecalis* QZ243 | FA2-2 with *acpA* expression plasmid | This work |
| *E. faecalis* QZ244 | *∆acpB* with *acpA* expression plasmid | This work |
| *E. faecalis* QZ426 | *∆fabN* with *fabZ* expression plasmid | This work |
| *E. faecalis* ZL53 | *∆fabI* | Zhu et al., 2013 |
| *E. faecalis* QZ431 | *∆fabN* with *acpA* expression plasmid | This work |
| *E. faecalis* QZ179 | FA2-2 with *L. lactis plsX* expression plasmid | This work |
| *E. faecalis* QZ180 | FA2-2 with *E. faecalis plsX* expression plasmid | This work |
| *E. faecalis* QZ423 | *∆fabN* with *fabN* expression plasmid | This work |
| *E. faecalis* QZ162 | *∆plsX* with *E. faecalis plsX* expression plasmid | This work |
| *E. faecalis* QZ522 | *∆fabT* with *L. lactis plsX* expression plasmid | This work |
| *E. faecalis* QZ523 | *∆fabT* with *E. faecalis plsX* expression plasmid | This work |
| *E. faecalis* QZ436 | *∆fabN* with *E. faecalis plsX* expression plasmid | This work |
| *E. faecalis* QZ469 | *∆fabN* with *L. lactis plsX* expression plasmid | This work |
| *E. faecalis* QZ479 | *∆fabN* with co-expression plasmid of *acpA* and *E. faecalis plsX* | This work |
| *E. faecalis* QZ494 | *∆fabN* with co-expression plasmid of *acpA* and *L. lactis plsX* | This work |
| *E. faecalis* QZ500 | FA2-2 with co-expression plasmid of *acpA* and *L. lactis plsX* | This work |
| *E. faecalis* QZ514 | FA2-2 with co-expression plasmid of *acpA* and *E. faecalis plsX* | This work |
| *E. faecalis* QZ501 | *∆acpB* with *L. lactis plsX* expression plasmid | This work |
| *E. faecalis* QZ502 | *∆acpB* with co-expression plasmid of *acpA* and *L. lactis plsX* | This work |
| *E. faecalis* QZ219 | FA2-2 with *lacZ* expression plasmid from *fabT* promoter | Zou et al., 2022 |
| *E. faecalis* QZ239 | FA2-2 with *lacZ* expression plasmid from *fabI* promoter | Zou et al., 2022 |
| *E. faecalis* QZ241 | FA2-2 with *lacZ* expression plasmid from *fabO* promoter | Zou et al., 2022 |
| *E. faecalis* QZ267 | *∆fabN* with *lacZ* expression plasmid from *fabT* promoter | This work |
| *E. faecalis* QZ438 | *∆fabN* with *lacZ* expression plasmid from *fabI* promoter | This work |
| *E. faecalis* QZ439 | *∆fabN* with *lacZ* expression plasmid from *fabO* promoter | This work |
|  |  |  |
| **Plasmids** |  |  |
| pQZ28 | Shuttled plasmid vector with a p32 promoter modified from pZL277 by replacing the chloramphenicol-resistant gene with erythromycin-resistant gene, *E. faecalis* expression | Zou et al., 2023 |
| pQZ43 | *E. faecalis plsX* in pQZ28 | Zou et al., 2023 |
| pDHJ544 | *L. lactis plsX* in pQZ28 | This work |
| pDHJ520 | *E. faecalis fabN* in pQZ28 | Dong & Cronan, 2022b |
| pQZ31 | *E. faecalis acpA* in pQZ28 | This work |
| pQZ422 | *E. faecalis fabZ* in pQZ28 | This work |
| pQZ214 | *E. faecalis fabT* start region (-389 to +35) at 5'-end of *lacZ* in pBHK322 | Zou et al., 2022 |
| pQZ235 | *E. faecalis fabI* start region (-297 to +35) at 5'-end of *lacZ* in pBHK322 | Zou et al., 2022 |
| pQZ238 | *E. faecalis fabO* start region (-297 to +35) at 5'-end of *lacZ* in pBHK322 | Zou et al., 2022 |
| pQZ472 | pQZ28 with *acpA* and *E. faecalis plsX* | This work |
| pQZ490 | pQZ28 with *acpA* and *L. lactis plsX* | This work |

**Table S2 Oligonucleotides primers used in the study**

| **Primers*** | **Sequence 5'-3'** |
| --- | --- |
| LlplsX F1 | GTAGGTAAAAAAATAAAAGGAGGTGAACCATGAAAATTGCAATTGATGCA |
| LlplsX R1 | AAACAGCTATGACATGATTACGAATTCACAGCTCAATTCTGTCAGTA |
| pQZ28 F1 | GAATTCGTAATCATGTCATAGCTGTTT |
| pQZ28-p32 R1 | GGTTCACCTCCTTTTATTTTTTTACCTAC |
| EfacpA NcoI F | CATGCCATGGTATTTGAAAAA |
| EfacpA EcoRI R | CCGGAATTCTTAGTTTGCTTGTTG |
| EffabZ NcoI F | CATGCCATGGCTATGAAATTAACAATTACAGAAATTCAA |
| EffabZ KpnI R | CGGGGTACCCTATCCAATCATAAAGGTTAATTCA |
| P32 F2 | AGGAAATAAGGATCCAGATTAATAGTTTTAGCTATTAATCTTTT |
| EfacpA R2 | TAATCTTTATTATTATTAGTTTGCTTGTTGTTTTTCT |
| P32 F3 | CAACAAGCAAACTAATAATAATAAAGATTAATAGTTTTAGCTATTAATCTTTT |
| EfplsX R | CATGATTACGAATTCTTACTCTGCTTTGCCTTCA |
| pQZ28 F2 | GGCAAAGCAGAGTAAGAATTCGTAATCATGTCATAGCT |
| pQZ28 R2 | TAAAACTATTAATCTGGATCCTTATTTCCTCCC |
| pQZ28 F3 | AAAATGGACAAATAAGAATTCGTAATCATGTCATAGCT |
| LlplsX R2 | CATGATTACGAATTCTTATTTGTCCATTTTTTCAAAATGT |

* The primer sequences were based on the *E. faecalis* V583 or L. lactis genome sequences. The underlined sequences indicate the restriction sites used in the study.


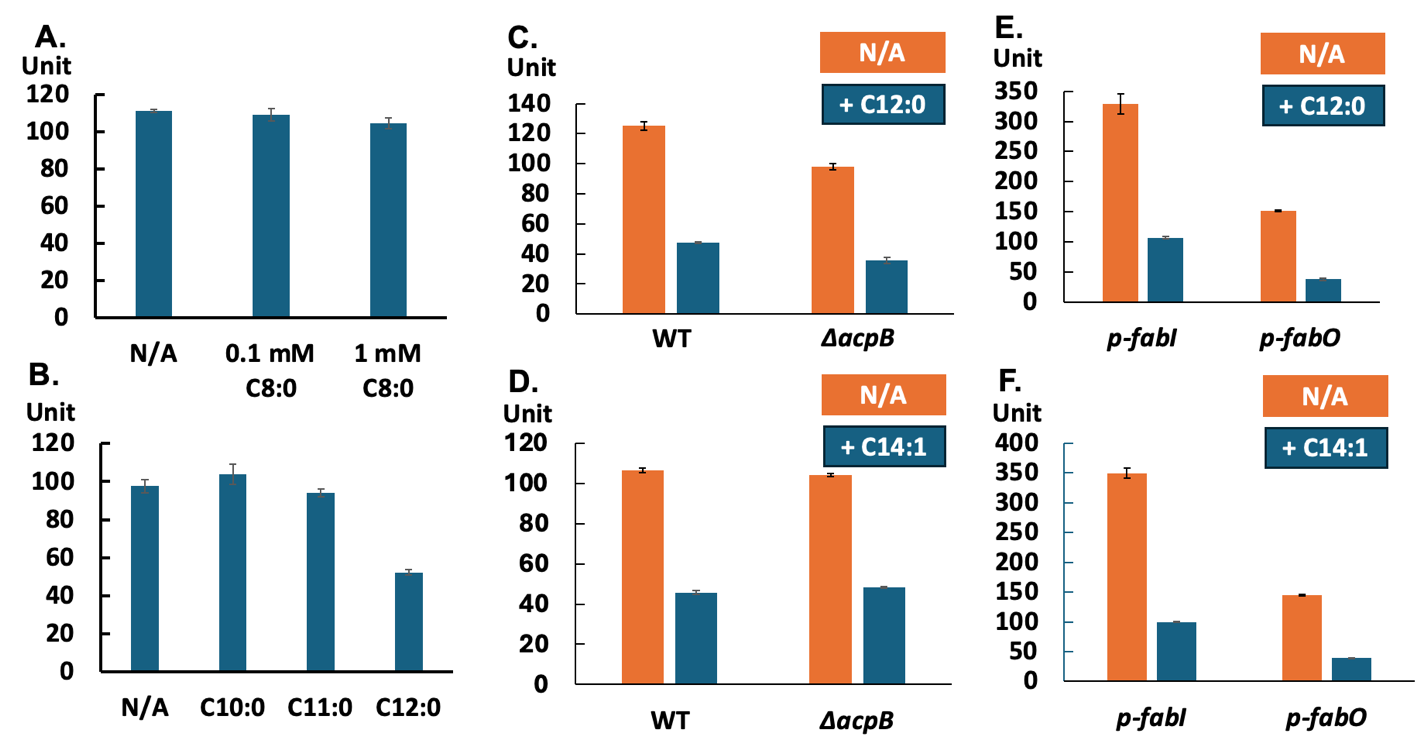


Figure S1. Effects of exogenous fatty acids on expression of *E. faecalis fab* genes. **A**. Expression of β-galactosidase from the *fabT* promoter in the *E. faecalis* wild-type strain cultured with different concentrations of octanoic acid (C8:0). **B**. Expression of β-galactosidase from the *fabT* promoter in *E. faecalis* wild-type strain in the presence of decanoic acid (C10:0), undecanoic acid (C11:0), or lauric acid (C12:0). **C-D**. Expression of β-galactosidase from the *fabT* promoter in *E. faecalis* wild-type and *∆acpB* strains in the presence of lauric acid (C12:0) (**C**) or *cis*-5 tetradecenoic acid (C14:1) (**D**). **E-F**. Expression of β-galactosidase from the *fabI* or *fabO* promoters in the *E. faecalis* wild-type strain in the presence of lauric acid (C12:0) (**E**) or *cis*-5 tetradecenoic acid (C14:1) (**F**).


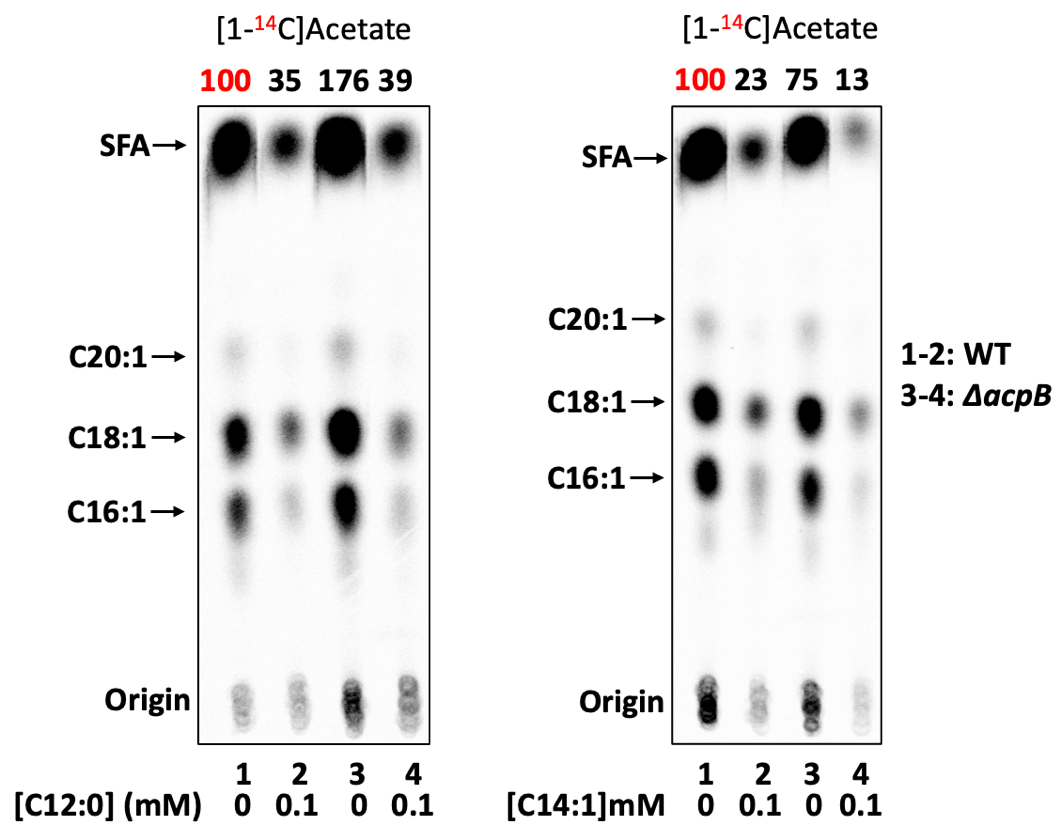


Figure S2: *De novo* synthesis of phospholipid fatty acyl chains in *E. faecalis* wild-type and *∆acpB* strains in the presence of lauric acid (C12:0) (left plate) or *cis*-5-tetradecenoic acid (C14:1) (right plate).


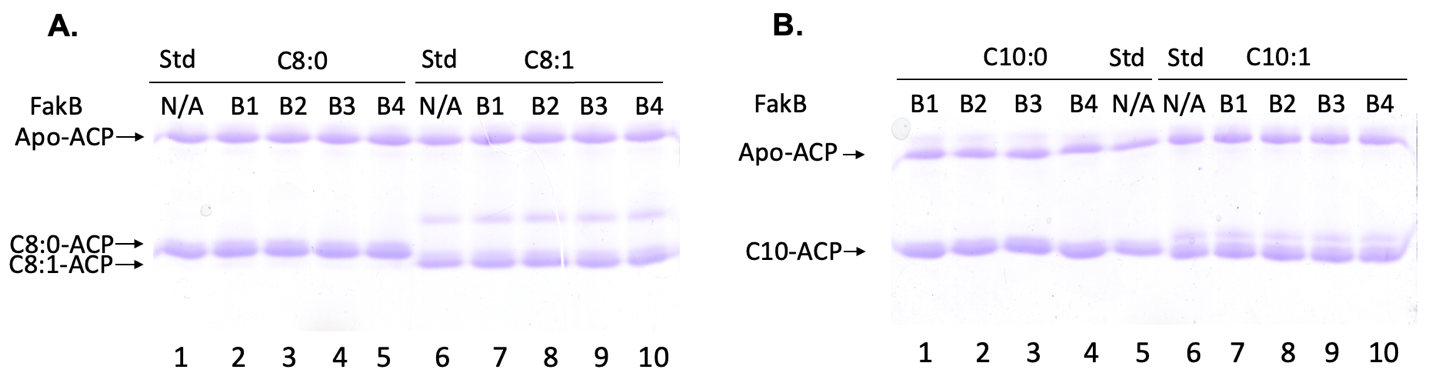


Figure S3. Activation and transfer of short chain intermediates of fatty acid synthesis by *E. faecalis* fatty acid kinase plus PlsX. **A**: Acylation of ACP by *trans*-2 octenoic acid (C8:1) through the Fak-PlsX reactions. **B**: Acylation of ACP by *trans*-2 decenoic acid (C10:1) catalyzed by the Fak-PlsX reactions. The *E. coli* enoyl-ACP standards were synthesized from free fatty acids by the AasS *Vibrio harveyi* acyl-ACP synthetase.


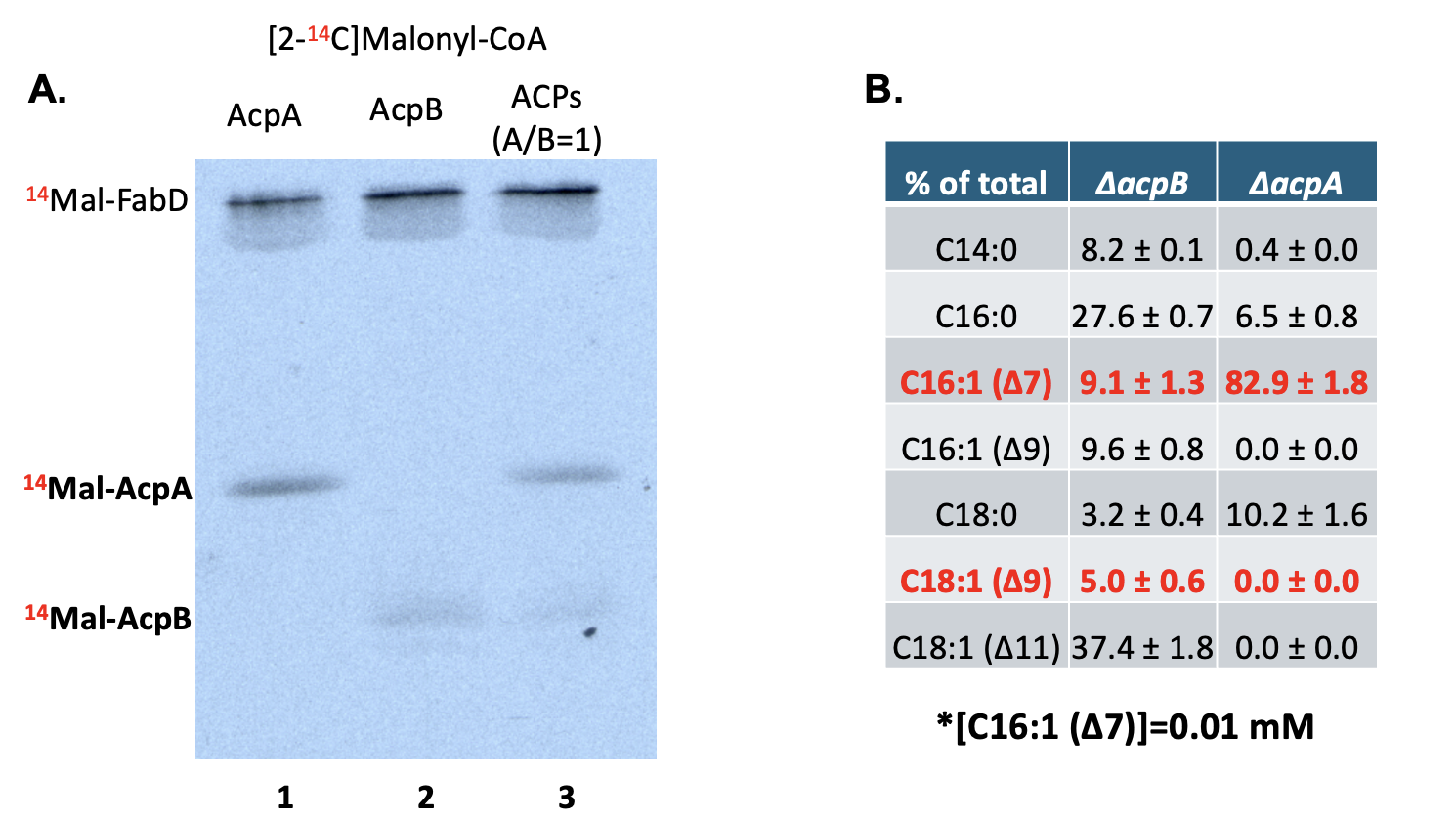


Figure S4. AcpA is essential for elongation of exogenous fatty acids. **A**. Malonylation of AcpA and AcpB by *E. faecalis* malonyl-CoA-ACP transacylase FabD. AcpA is a better substrate than AcpB in malonyl transferring from malonyl-CoA to ACP by *E. faecalis* FabD as expected from the inability of AcpB to replace AcpA (Dong Cronan, 2022). The bands were quantitated by ImageQuant software analyses of scans of the X-ray film. In lanes 1 and 2 the level of malonyl-AcpA was 2.7-fold greater than that of malonyl-AcpB. In lane 3 in which the two ACPs competed for malonyl-transfer, label in malonyl-AcpA exceeded that in malonyl-AcpB by 5-fold. **B**. Incorporation and elongation of *cis*-7-hexadecenoic acid (C16:1) by *E. faecalis ∆acpA* and *∆acpB* strains. In the *∆acpA* strain note the lack of elongation of *cis*-7-hexadecenoic acid to oleic acid (*cis*-9 C18:1) and the absence of the *de novo* synthesized unsaturated acids C16∆9 (palmitoleate) and C18∆11 (*cis*-vaccenate). These data indicate that the other fatty acids were incorporated from the medium.


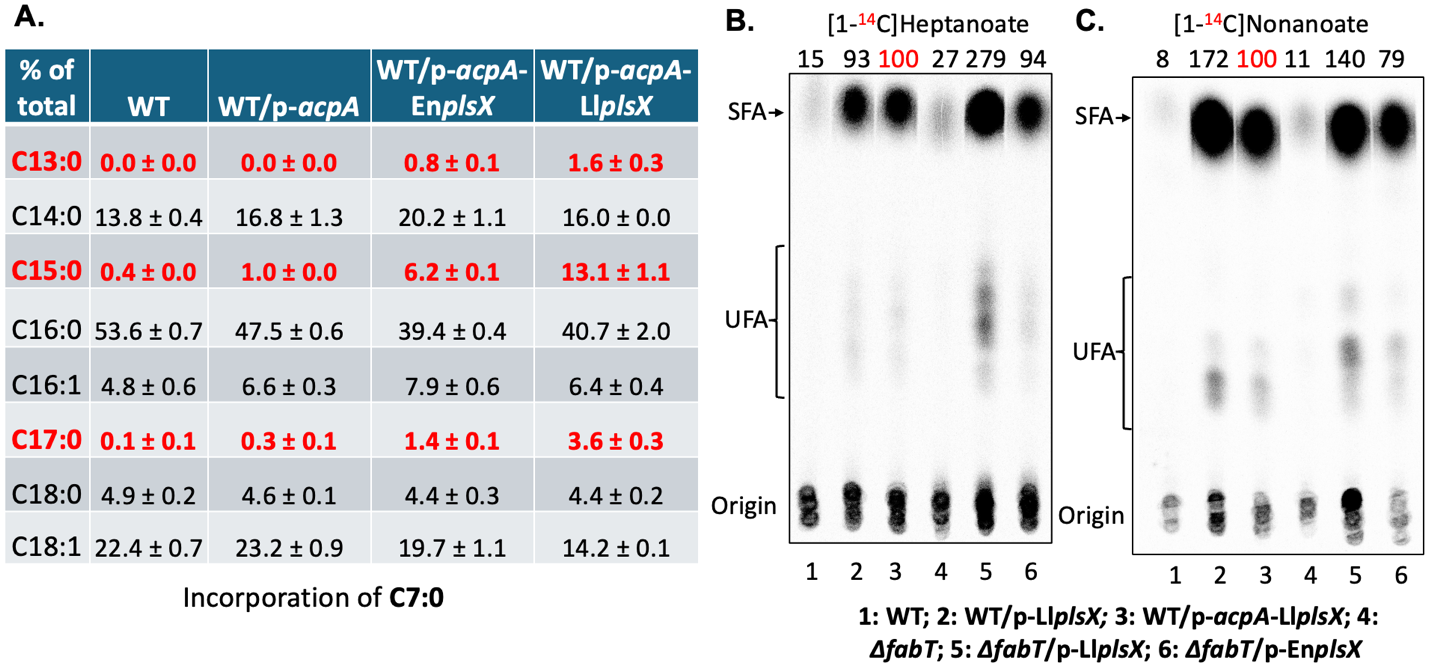


Figure S5. Coordination between FAS II and PlsX is beneficial for *E. faecalis* incorporation and elongation of short chain fatty acids. **A**. GC-MS analysis for incorporation and elongation of heptanoic acid (C7:0) by *E. faecalis* wild-type strain overexpressing AcpA coupled with *E. faecalis* PlsX overexpression or *L. lactis* PlsX expression. The acquired fatty acyl methyl esters were submitted for dimethyl sulfide (DMSD) treatment and then processed for mass spectrum analysis. **B-C**. Incorporation of [^14^C]heptanoate (left plate image) or [^14^C]nonanoate (right plate image) by the *E. faecalis* *∆fabT* strain expressing *L. lactis* PlsX or overexpressing *E. faecalis* PlsX. The numbers above the lanes are the radioactive label incorporation values relative to the value (100) for the wild-type strain overexpressing AcpA coupled with *L. lactis* PlsX expression. In panel A, heptanoic acid was supplied at 1 mM.


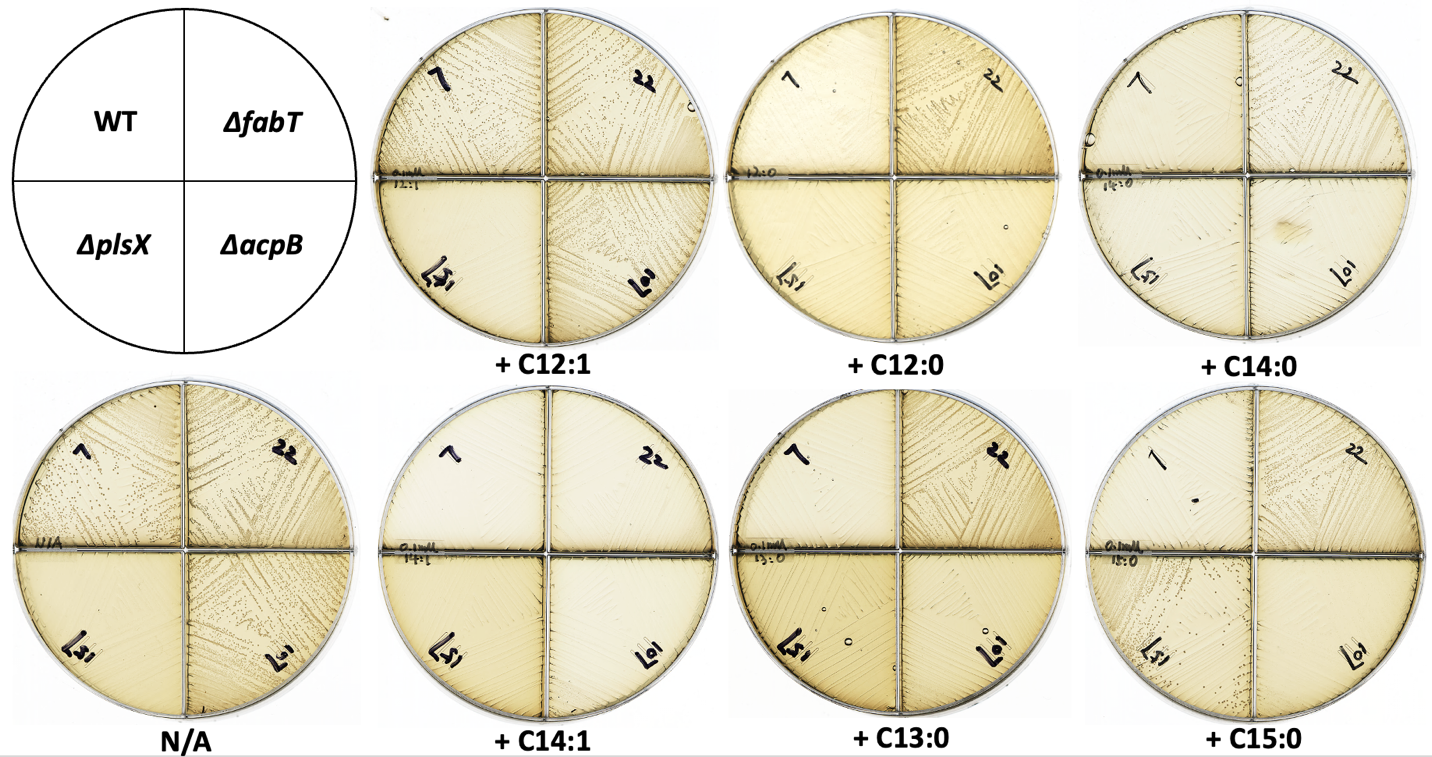


Figure S6. Growth of several *E. faecalis* strains in the presence of unsaturated fatty acids *cis*-5-dodecenoic acid (C12:1) or *cis*-5-tetradecenoic acid (C14:1), or the saturated acids, lauric acid (C12:0), tridecanoic acid (C13:0), myristic acid (C14:0) or pentadecanoic acid (C15:0). Since low levels of lauric acid can be directly incorporated into phospholipid without elongation, reverse phase thin layer chromatography of the methyl esters was used to demonstrate elongation of laurate to C14 and C16 chains (data not shown).


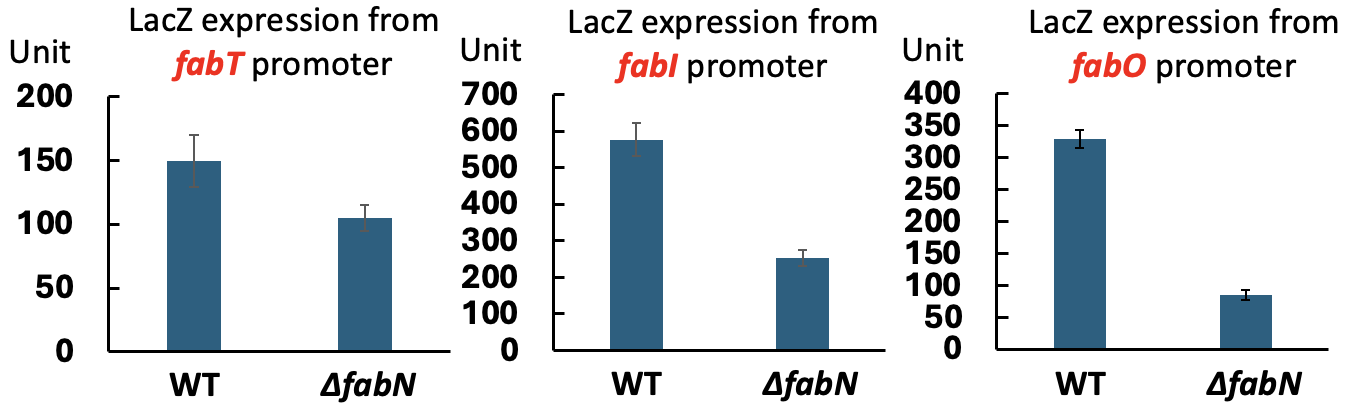


**+ 0.01 mM C18:1**

Figure S7. Expression of β-galactosidase from the *fabT* (left), *fabI* (middle), or *fabO* (right) promoters in the *E. faecalis ∆fabN* strain in the presence of 0.01 mM *cis*-vaccenic acid (*cis*-11 C18:1).


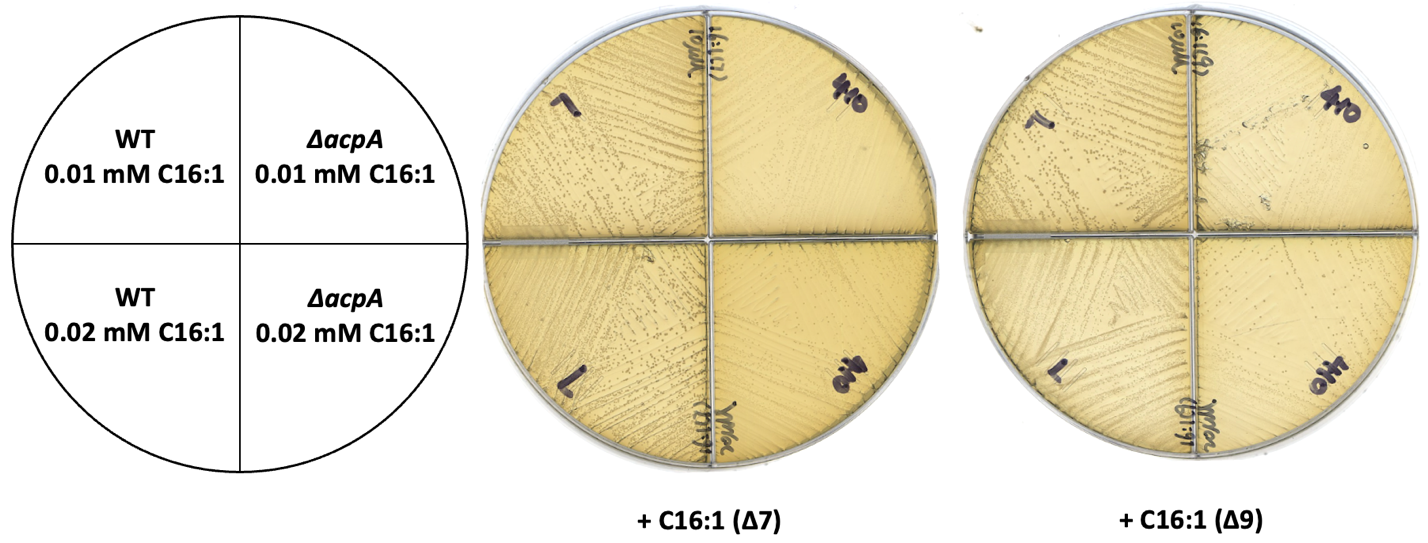


Fig. S8. Growth of *E. faecalis* wild-type strains in the presence of low concentration of unsaturated *cis*-C16 fatty acids C16∆7 and C16∆9 (palmitoleic) acids. At low concentrations these acids also allow weak growth of the *∆acpA* strain as shown.
